# Abstracts

**DOI:** 10.1111/jdi.12670

**Published:** 2017-05-05

## Oral Presentation Abstracts

### AI‐5‐1

#### Indium‐labeled Exendin probe enables to quantify beta cell mass non‐invasively with SPECT imaging

**N. Fujita^1^, H. Fujimoto^1^, K. Hamamatsu^1^, T. Murakami^1^, H. Kimura^2^, K. Toyoda^1^, H. Saji^3^ and N. Inagaki^1^**

^1^Department of Diabetes, Endocrinology and Nutrition, Graduate School of Medicine, Kyoto University, Kyoto, Japan, ^2^Department of Analytical and Bioinorganic Chemistry, Kyoto Pharmaceutical University, Kyoto, Japan, ^3^Department of Patho‐Functional Bioanalysis, Graduate School of Pharmaceutical Sciences, Kyoto University, Kyoto, Japan

We aim to establish a non‐invasive method to quantify beta cell mass (BCM) by SPECT with indium‐labeled Exendin probe. We confirmed specific accumulation to beta cells by autoradiography with pancreatic sections of MIP‐GFP mice. We took SPECT imaging on 8 NOD mice after injecting the probe intravenously. Following the SPECT, we harvested mice’ pancreas and calculated BCM from immuno‐stained sections. On MIP‐GFP mice’ pancreatic sections, fluorescent signals corresponded to radioactive signals, and the intensity of both signals significantly correlated. BCM significantly correlated to SPECT signal from pancreas. The probe specifically accumulated to islets and BCM can be non‐invasively quantified with the probe without harvesting pancreas.

### AI‐5‐2

#### Role of human urine‐derived stem cells in the treatment of diabetic mice

**T. Zhao^1,2^, Y. Sun^1,2^, D. Luo^1,2^, X. Niu^3^, H. Zhu^1,2^, Y. Wang^3^ and C. Wang^1,2^**

^1^Shanghai Diabetes Institute, Shanghai Jiao Tong University, Shanghai, China, ^2^Department of Endocrinology and Metabolism, Shanghai Jiao Tong University Affiliated Sixth People's Hospital, Shanghai, China, ^3^Institute of Microsurgery on Extremities, Shanghai Jiao Tong University Affiliated Sixth People's Hospital, Shanghai, China

This study was to explore the effects of human urine‐derived stem cells (USCs) in treating animal models of diabetes. USCs isolated from the voided urine were transplanted into STZ‐treated nude mice. USCs expressed mesenchymal stem cell markers and could differentiate into the bone and fat in vitro. Transplantation of USCs into high‐dose STZ treated nude mice resulted in longer median survival time,and low‐dose STZ treated ones showed improved glucose tolerance. After the exendin‐4,activin‐A and niacinamide induction,USCs presented with dithizone positive staining, glucagon and PDX‐1 mRNA expression. Taken together, transplantation of USCs may be useful for diabetes treatment.

The study was supported by Shanghai Nature Science Fund (16ZR1425800).

### AI‐5‐3

#### LAF237 enhances glucose tolerance and graft β‐cell mass in diabetic mice transplanted with sufficient numbers of islets

**J.‐H. Juang^1^, C.‐Y. Chen^1^, C.‐W. Kao^1^, C.‐H. Kuo^1^ and C.‐T. Chen^2^**

^1^Division of Endocrinology and Metabolism, Chang Gung University and Memorial Hospital, Taiwan, ^2^Institute of Biotechnology and Pharmaceutical Research, National Health Research Institutes, Taiwan

We syngeneically transplanted 150 or 300 islets to each diabetic mouse, and then treated recipients with distilled water or LAF237 for 6 weeks. In mice transplanted with 150 islets, there were no differences between LAF237‐treated and control group in glucose tolerance test (GTT) at 2, 4 and 6 weeks and the graft β‐cell mass at 6 weeks. However, in mice transplanted with 300 islets, the GTT was lower in the LAF237‐treated group than controls at 4 and 6 weeks. At 6 weeks, LAF237‐treated group had more graft β‐cell mass than controls. Our results indicate posttransplant LAF237 treatment improves glucose tolerance and expands graft β‐cell mass in diabetic recipients transplanted with a sufficient but not marginal number of islets.

### AI‐5‐4

#### Suppression of ROS production by Exendin‐4 in PSC attenuates the high glucose‐induced islet fibrosis

**J. Kim, Y.‐H. You and K.‐H. Yoon**

Department of Endocrnology, The Catholic University of Korea

Ex‐4 significantly reduced Ang II and TGF‐β1 production by inhibition of ROS production but not ERK phosphorylation. This inhibitory effect of Ex‐4 was largely related with cAMP/PKA signaling pathway. Thus these results suggest that Ex‐4 may be useful not only as an anti‐diabetic agent but also as an anti‐fibrotic agent in type 2 diabetes.

### AI‐5‐5

#### Impaired insulin secretion and severe glucose intolerance caused by deleting β‐cell specific KATP channels

**S. Okechi Oduori^1^, K. Minami^1^, N. Yokoi^1^, H. Takahashi^1^, Y. Maejima^2^, K. Shimomura^2^, L. Pedro Herrera^3^ and S. Seino^1^**

^1^Molecular and Metabolic Medicine, Kobe University Graduate School of Medicine, ^2^Department of Electrophysiology and Oncology, Fukushima Medical University, Fukushima, Japan, ^3^Faculty of Medicine, University of Geneva, Geneva, Switzerland

We generated β‐cell KATP knockout (βKir6.2 KO) mice to investigate the role of the β‐cell KATP channel in insulin secretion and glucose homeostasis. βKir6.2 KO mice had elevated fasting blood glucose level and their tolerance to both oral glucose and meal ingestion was severely impaired. Glucose and meal induced insulin secretion and GIIS in isolated islets were severely impaired, but βKir6.2 KO islets had elevated basal insulin level. Glibenclamide had no hypoglycemic effect yet liraglutide completely normalized glucose tolerance and insulin secretion. GIP level in βKir6.2 KO mice was significantly elevated. Thus incretin signaling is enhanced in βKir6.2 KO mice, to possibly compensate for the impaired insulin response to glucose.

### AI‐5‐6

#### Effects of β‐cell function related genetic variants on predicting deterioration of glucose tolerance in the Chinese

**J. Yan, F. Jiang, C. Hu and W. Jia**

Shanghai Diabetes Institute, Shanghai Jiao Tong University Affiliated Sixth People's Hospital

We aimed to investigate the predicted effect of IS‐GRS and IR‐GRS on deterioration of glucose tolerance. We genotyped 89 T2D SNPs in NGT individuals (*N* = 3412) and categorized them into insulin secretion (IS) or insulin resistance (IR) related loci. IS‐GRS and IR‐GRS were constructed to assess their predicting effects in a community‐based 9‐year prospective cohort (*N* = 2495) in the Chinese. IS‐GRS predicted incidence of T2D and IGR in the logistic regression (OR 0.945, 95% CI 0.911, 0.981, *P* = 0.0031) as well as in the Cox model (HR 0.968, 95% CI 0.939, 0.999, *P* = 0.0399) after adjusting for age, sex, body mass index (BMI) at baseline during the 9‐year follow‐up. IS‐GRS could predict deterioration of glucose tolerance in the Chinese population.

### AI‐6‐1

#### Euglycemia by long‐term insulin pump therapy differentially improve C‐peptide or disposition index in type 2 diabetes

**S. Choi^1^, E. Hong^1^ and Y. Noh^2^**

^1^Department of Internal Medicine, Konkuk University School of Medicine, ^2^Department of Biochemistry, Konkuk University School of Medicine

We applied insulin pump therapy to 163 type 2 diabetic patients for 4 years. C‐peptidogenic Index (CI), Matsuda Index (MI),and disposition Index (DI) were calculated after mixed meal ingestion.

Patients were grouped into high MI [insulin sensitive (IS) ] group and low MI [insulin resistant (IR)] group by the mean value of baseline MIs. HbA1c decreased significantly from 8.9% to 6.6% in both groups. In the IS group, serum C‐peptide increased significantly but DI did not change during the 4‐years CSII therapy. Whereas, in the IR group, serum C‐peptide did not increase but the DI increased significantly. Therefore, achieving near euglycemia using long‐term insulin pump therapy seems to restore the original defect in each diabetic group.

### AI‐6‐2

#### Safety of metformin therapy and serum lactate level in T2DM living on an oxygen‐deficient plateau, Tibet, China

**Q. Ren^1^, X. Lv^1^, L. Zhou^2^, Y. Geng^1^, J. Song^1^, A. Mina^1^, B. Puchi^1^, S. Yang^1^, S. Meng^1^ and L. Yang^1^**

^1^Department of Endocrinology and Metabolism, People's Hospital of Tibet Autonomous Region^2^Department of Endocrinology and Metabolism, Peking University People's Hospital

The absolute risk of lactic acidosis in patients treated with metformin appears to be low in the general population. However, in the Tibetan plateau, an extreme oxygen‐deficient environment for human beings, there are no data available concerning the safety of metformin in the treatment of patients with T2DM. Using data of 166 inhabitants of the Tibetan plateau region, which is an oxygen‐deficient environment, we found treatment with metformin did not increase the risk of hyperlactacidemia or lactate acidosis. The level of lactate was significantly associated with FBG and HbA1c levels.

### AI‐6‐3

#### Renoprotective effect of liraglutide in Japanese patients with type 2 diabetes enrolled in dialysis prevention program

**Y. Hamamoto, N. Kitatani, F. Koshian, N. Makabe, H. Kuwata, K. Watanabe, T. Hyo, N. Tanaka, T. Kurose and Y. Seino**

Center for Diabetes, Endocrinology and Metabolism, Kansai Electric Power Hospital

Liraglutide (Lira) may have renoprotective effect. We examined the effect of Lira on renal function in T2D patients who were in dialysis prevention program (DPP). A total of 20 (male:15) patients initiated Lira between Apr 2014 – Jul 2015 were retrospectively identified from database. Changes in eGFR, HbA1c, body weight, blood pressure (BP), and ACR were examined 1‐year prior and after Lira. Mean eGFR was 70.1 ± 17.4 mL/min/1.73 m^2^. ARB/ACEI and statin were given in 80% and 70%. Whereas eGFR decreased before Lira, it tended to increase after Lira (−2.82 ± 6.55 vs. 2.17 ± 7.12 mL/min/1.73 m^2^/year, *P* = 0.067). HbA1c, BP and ACR did not change. In conclusion, Lira may improve eGFR even in patients who are on ARB/ACEI and statin and treated in DPP.

### AI‐6‐4

#### Efficacy and safety of a fixed‐ratio combination of basal insulin and GLP‐1 receptor agonist in T2DM patients

**X. Gao, X. Cai, W. Yang, Y. Chen and L. Ji**

Department of Endocrinology and Metabolism, Peking University People's Hospital

Totally 8 studies were included in this meta‐analysis. A fixed‐ratio combination of basal insulin and GLP‐1RA showed a significant HbA1c reduction, fasting plasma glucose and postprandial glucose reduction, body weight reduction from baseline. Compared with basal insulin, the fixed‐ratio combination therapy led to significant decreases in HbA1c and body weight, but a comparable decrease in FPG and a comparable incidence of hypoglycemia. Compared with GLP‐1RA, the fixed‐ratio combination therapy led to significant decreases in HbA1c and FPG, but a lower decrease in body weight and a higher rate of hypoglycemia.

In a word, a fixed‐ratio combination of basal insulin and GLP‐1RA was superior to its components given alone in glycemic control.

### AI‐6‐5

#### SGLT2 inhibitor luseogliflozin use and dietary carbohydrate intake in Japanese individuals with type 2 diabetes

**T. Haraguchi^1^, D. Yabe^1,2,3,4^, M. Iwasaki^2,3^, H. Kuwata^1,2^, Y. Hamamoto^1,2,3^, T. Kurose^1,2^, K. Sumita^5^, H. Yamazato^5^, S. Kanada^5^ and Y. Seino^1,2^**

^1^Center for Diabetes, Endocrinology and Metabolism, Kansai Electric Power Hospital, Osaka, Japan^2^Yutaka Seino Distinguished Center for Diabetes Research, Kansai Electric Power Medical Research Institute, Kobe, Japan^3^Center for Metabolism and Clinical Nutrition, Kansai Electric Power Hospital, Osaka, Japan^4^Division of Molecular and Metabolic Medicine, Department of Physiology and Cell Biology, Kobe University Graduate School of Medicine, Kobe, Japan^5^Medical Corporation Heishinkai OCROM Clinic

**Methods**

Individuals with type 2 diabetes (T2D) were randomly assigned to three different carbohydrate‐adjusted meals for 14 days[HC‐HGI, 55% of total energy from carbohydrate (TEC) and high glycemic index (GI); HC‐LGI, 55% TEC and low GI; and LC‐HGI, 40% TEC and high GI]. All subjects received luseogliflozin 2.5 mg once daily for the last 7 days.

**Results**

Ketone bodies on Day 15 were significantly increased in LC‐HGI compared to HC‐HGI and HC‐LGI.

**Conclusion**

This study shows that SGLT2 inhibitor luseogliflozin has similar efficacy and safety in Japanese individuals with T2D when meals contain 40–55% TEC, but a strict low‐carbohydrate diet on this class of drug should be avoided to prevent SGLT2 inhibitor‐associated diabetic ketoacidosis.

### AI‐6‐6

#### Safety, tolerability and pharmacokinetics of Imeglimin in healthy Japanese subjects

**S. Bolze, S. Dauchy, C. Tang, P. Fouqueray and Y. Itoh**

POXEL

Imeglimin (IME) is a new glucose‐lowering agent improving insulin secretion and sensitivity. Phase 2b in Japan is ongoing. IME safety, tolerability and pharmacokinetics (PK) were assessed in Japanese healthy volunteers (HV) after repeated doses (RD) up to 2000 mg. The maximum tolerated dose (MTD) was assessed in Japanese and Caucasian HV after single doses (SD) up to 8000 mg. After RD IME exposure increased with dose up to 1500 mg with good tolerance. After SD exposure increased less than dose‐proportional and tolerance was good up to 6000 mg considered as the MTD in both populations. Overall the safety and PK of IME were similar in both Japanese and Caucasian populations.

### AI‐6‐7

#### Impaired glucagon‐like peptide 1 response is associated with metabolic syndrome in patients with type 2 diabetes

**S. Yoo^1^, E.‐J. Yang^2^, S. A. Lee^1,2^ and G. Koh^1,2^**

^1^Department of Internal Medicine, Jeju national University Hospital^2^Department of Internal Medicine, Jeju National University School of Medicine

We measured intact GLP‐1 (iGLP‐1) and intact GIP (iGIP) levels in a fasted state and 30 min after ingestion of a standard mixed meal in 334 patients with type 2 diabetes (T2D). The ΔiGLP‐1 was significantly lower in those with metabolic syndrome (MetS). The ΔiGLP‐1 levels decreased significantly as the number of MetS components increased. In a hierarchical logistic regression analysis, the ΔiGLP‐1 level was found to be a significant contributor to MetS. Taken together, the impaired iGLP‐1 response is independently associated with MetS in T2D patients.

### AI‐6‐8

#### Neck circumference and waist circumference identify metabolic syndrome with similar efficacy

**Y. Luo^1^, X. Ma^1^, Y. Shen^1^, Y. Xu^1^, Q. Xiong^1^, X. Zhang^1^, Y. Xiao^2^, Y. Bao^1^ and W. Jia^1^**

^1^Department of Endocrinology and Metabolism, Shanghai Jiao Tong University Affiliated Sixth People's Hospital, Shanghai Clinical Center for Diabetes, Shanghai Key Clinical Center for Metabolic Disease, Shanghai Diabetes Institute, Shanghai Key Laboratory^2^Department of Radiology, Shanghai Jiao Tong University Affiliated Sixth People's Hospital

Neck circumference (NC) is a new anthropometric index for estimating obesity. We aimed to determine the relationship between NC and body fat content and distribution as well as the efficacy of NC for identifying metabolic disorders. In 1943 subjects (783 men, 1160 women) with a mean age of 57.9 ± 7.3 years, visceral fat content was independently correlated with NC. A NC value of 38.5 cm for men and 34.5 cm for women were the optimal cutoffs for identifying visceral obesity. There were no statistically significant differences between the proportions of metabolic disorders identified by an increased NC and waist circumference. Therefore, NC has the same power as waist circumference for identifying metabolic disorders in a Chinese population.

### AI‐6‐9

#### Relationship between vitamin D status, metabolic syndrome and incident diabetes: A prospective cohort study

**E. Y. Lee, Y. Yang, H.‐S. Kim, S.‐H. Lee, J. H. Cho, B. Y. Cha and K.‐H. Yoon**

Department of Internal Medicine, College of Medicine, The Catholic University of Korea

**Aim**

We investigated the association of low serum 25(OH)D levels with incident diabetes and its combined effects with metabolic syndrome (MetS) in Korean population.

**Methods**

We studied 1,038 subjects aged ≥40 years and diabetes was diagnosed with 75 g oral glucose tolerance test.

**Results**

During 4 year follow‐up, incident diabetes was developed in 161 (18.4%) subjects. Low serum 25(OH)D levels alone was not associated with incident diabetes. However, subjects with low 25(OH)D and MetS had significantly increased odds for incident diabetes when compared to those with high 25(OH)D and without metabolic syndrome.

**Conclusions**

Low serum 25(OH)D levels were associated with incident diabetes only when it was combined with MetS.

### AI‐6‐10

#### Xanthine oxidase activity in plasma reflects systemic insulin resistance in patients with T2DM and metabolic syndrome

**S. Sunagawa^1^, Y. Murayama^1^, M. Nakandakari^1^, T. Ikematsu^1^, C. Koduka^1^, T. Ikema^1^, T. Sirakura^2^, M. Tamura^2^, M. Shimabukuro^3^ and H. Masuzaki^1^**

^1^Division of Endocrinology, Diabetes and Metabolism, Hematology, Rheumatology (Second Department of Internal Medicine), Graduate School of Medicine, University of the Ryukyus^2^Teijin Pharma Limited^3^Division of Diabetes, Endocrinology and Metabolism, Fukushima medical University

**Background**

It has been recognized that oxidative stress produced by xanthine oxidase (XO), a key enzyme in uric acid production, would be related with vascular endothelial dysfunction in patients with metabolic syndrome (MetS).

**Methods**

We enrolled 60 patients with T2DM and MetS (BMI over 25) and we consecutively measured plasma XO activity.

**Results**

Plasma XO activity was correlated positively with fasting FIRI (r = 0.82 *P* < 0.01) and HOMA‐R (r = 0.68 *P* < 0.01). In contrast, plasma XO activity was not correlated with values of ages, HbA1c, BMI and serum uric acid.

**Conclusion**

Plasma XO activity may serve as a novel surrogate marker to evaluate the risks for insulin resistance and atherosclerosis influenced by systemic oxidative stress.

### AI‐6‐11

#### Clinical prospects of altered mRNA expression levels of Matrix Metalloprotienases 1, 2 and 9 in metabolic syndrome

**S. Y. Singh^1,2^, P. S. Kumar^1^, M. S. Kumar^3^, K. Usman^4^ and S. Khattri^2^**

^1^Department of VARD‐ Human Health, National Innovation Foundation, Ahmedabad, Gujarat, India^2^Department of Pharmacology, King George's Medical University, Lucknow, UP, India^3^Department of Biochemistry, MLN Medical College, Allahabad, UP, India^4^Department of Medicine, King George's Medical University, Lucknow, UP, India

This case control study emphasized on the effect of impaired expression of genes MMP 1,2,9 in metabolic syndrome (MetS) and their correlation with clinical factors. A significantly (*P* < 0.001) higher serum MMP‐2 (39.13 ± 19.96 ng/mL) and MMP‐9 (73.93 ± 33.59 ng/mL) levels were detected in cases. Expression levels of MMP‐2 (1.39 ± 0.26) and MMP‐9 (1.82 ± 0.41) in cases were significantly higher than controls (0.73 ± 0.17 and 0.71 ± 0.19 respectively, *P* < 0.05). There was no significant difference detected in MMP‐1 expression. Expression levels of MMP‐2 and MMP‐9 was significantly correlated with clinical factors of MetS. Clinical implication of results may help to identify the individuals with high risk of MetS and further complications.

### AI‐6‐12

#### Central obesity measured by Controlled Attenuation Parameter is more important factor to predict significant steatosis

**J. H. Lee^1^ and K. J. Kim^2^**

^1^Department of Internal Medicine, SEONAM University college of Medicine, Myongji Hospital^2^Department of Internal Medicine, yonsei University college of Medicine

Central obesity measured by CAP is more important factor to predict significant steatosis

The central obesity significantly contribute to occurrence of hepatic steatosis, but the relationship between CAP value and VFA by CT is not investigated. A total of 304 subjects were enrolled prospectively. Multivariate linear regression analysis revealed that VFA was significantly related with CAP. In the multiple logistic regression analysis, VFA and TG were independent risk factor for significant hepatic steatosis. Patients with a higher VFA were at a greater risk of significant hepatic steatosis. Our data suggested that surveillance of hepatic steatosis need to be performed according the parameter which represents central obesity, not just BMI.

### AI‐6‐13

#### Association between free triiodothyronine levels and nonalcoholic fatty liver disease in euthyroid Chinese

**P. Chen^1,2,3,4,5^, X. Hou^1,2,3,4,5^, S. Chen^1,2,3,4,5^, J. Wu^1,2,3,4,5^, Y. Bao^1,2,3,4,5^ and W. Jia^1,2,3,4,5^**

^1^Department of Endocrinology and Metabolism, Shanghai Jiao Tong University Affiliated Sixth People's Hospital^2^Shanghai Clinical Center for Diabetes^3^Shanghai Diabetes Institute^4^Shanghai Key Laboratory of Diabetes Mellitus^5^Shanghai Key Clinical Center for Metabolic Disease

**Objective**

To investigate the association between thyroid function and nonalcoholic fatty liver disease (NAFLD) in euthyroid Chinese in a community based population.

**Methods**

We included 9372 subjects with normal thyroid function in a cross‐sectional survey.

**Results**

4166 met the diagnosis criteria of NAFLD. The increasing trends in prevalence of NAFLD were observed along with elevated free triiodothyronine (FT3) quartiles. Multivariable logistic regression showed that higher FT3 levels were associated with prevalence of NAFLD, (OR (95%CI): 1.58 [1.39–1.80] for men and 1.75 [1.57–1.95] for women), While thyroid stimulating hormone and free thyroixine were not.

**Conclusion**

High normal FT3 levels were positively associated with prevalence of NAFLD.

### AI‐6‐14

#### Lower 25‐hydroxy vitamin D level was associated with epicardial fat thickness and NAFLD disease in metabolic syndrome

**E. Kim^1^, S. Y. Oh^1^, M. J. Lee^1^, J. H. Kim^1^, Y. K. Jeon^1^, B. H. Kim^1,2^ and I. Kim^1,2^**

^1^Department of Internal Medicine, Pusan National University College of Medicine^2^Department of Internal Medicine, Pusan National University Hospital and Biomedical Research Institute

The aim of this study was to determine if, and to what extent, epicardial fat thickness (EFT) and non‐alcoholic fatty liver disease (NAFLD) are related to vitamin D deficiency in patients with metabolic syndrome (MetS). A total of 1,162 consecutive patients in health screening center who underwent abdominal ulasrasonography, echocardiography and measurement of vitamin D levels were enrolled. EFT was significantly higher in patients with NAFLD compared with group without NAFLD. Patients in the first quartile of 25‐(OH) vitamin D level had a greater occurrence of NAFLD (odds ratio (OR) 3.17 [confidence interval (CI) 2.19–4.59]; *P* < 0.001), MetS (OR 2.38 [CI 1.57–3.60]; *P* < 0.001) than those in the fourth quartile.

### AI‐6‐15

#### Prevalence and risk factors for hyperlipidemia and hyperglycemia among Filipinos with HIV in a government treatment hub

**M. G. Yu^1^, C. Francisco^2^, E. Gonzales^3^, M. Alejandria^2^, E. M. Salvana^2^, P. F. Reganit^3^, P. Maningat^1^ and O. Sison^4^**

^1^Section of Endocrinology, Diabetes, and Metabolism, Department of Medicine, Philippine General Hospital^2^Section of Infectious Diseases, Department of Medicine, Philippine General Hospital^3^Section of Cardiology, Department of Medicine, Philippine General Hospital^4^Department of Clinical Epidemiology, College of Medicine, University of the Philippines Manila

**Methods**

We reviewed 635 Filipino HIV cases from 2004 to 2016, using multivariate regression to determine risk factors for hyperlipidemia and hyperglycemia before and after antiretroviral (ARV) use.

**Results**

In ARV‐naïve patients, male sex, hyperglycemia, and ARV delay>320 days from diagnosis were risk factors for hyperlipidemia, while hyperlipidemia was a risk factor for hyperglycemia. After ARV initiation, hyperglycemia, ARV use>36 months, WHO Stage 4 HIV, and an efavirenz‐based regimen were risk factors for hyperlipidemia, while age>30, hyperlipidemia, overweight BMI, and a zidovudine‐based regimen were risk factors for hyperglycemia.

**Conclusion**

Various factors contribute to hyperlipidemia and hyperglycemia among Filipino HIV cases.

### AII‐5‐1

Withdrawn.

### AII‐5‐2

#### Effectiveness assessment for the treatment and management platform of obesity in Korean child and youth

**S.‐Y. Lim^1^, J.‐H. Lee^1^, B. Kang^2^, E.‐Y. Lee^2^, Y. Yang^2^, Y.‐H. Choi^2^, B.‐K. Suh^3^ and K.‐H. Yoon^2^**

^1^Catholic Institute of U‐healthcare, The Catholic University of Korea^2^Department of Endocrinology and Metabolism, College of Medicine, The Catholic University of Korea^3^Department of Pediatrics, College of Medicine, The Catholic University of Korea

We aim to assess the effectiveness of the improvement in BMI percentile by applying integrated platform developed to treat and manage of childhood obesity in local community. This study was performed over 12‐months as a randomized clinical trial. A total of 154 subjects randomly assigned to two groups at baseline. Of those, 71 study subjects followed up were included for analysis. A 2 by 2 mixed model ANOVA was done to compare the change of BMI percentile. There was a significant interaction between group and time (*P* = 0.045). In intervention group, BMI percentile significantly decreased from 93.1 to 90.9. These findings show that effective management and treatment of childhood obesity will be available by using platform in local community.

### AII‐5‐3

#### Mobile based nutritional management tool for pregnant women with diabetes mellitus in Korea

**S.‐Y. Lim^1^, H.‐J. Yoo^1^, C.‐J. Han^1^, S.‐Y. Ko^1^, Y.‐H. Choi^2^, K.‐H. Yoon^2^ and J.‐H. Lee^1^**

^1^Catholic Institute of U‐healthcare, The Catholic University of Korea^2^Department of Endocrinology and Metabolism, College of Medicine, The Catholic University of Korea

The aim of this study was to develop the mobile based nutritional management tool can be designed for supporting self‐nutritional management for pregnant women with diabetes and evaluate feasibility. Mobile application as management tool of subject's diet has four main functions which are as follows. (1) Assessment appropriate maternal weight gain for gestational week, (2) Prescription total calories considering pre‐pregnancy weight and gestational week. And Suggestion of a recommended diet, (3) Providing nutritional information (4) Expert's evaluation of dietary records, user‐entered. This application was meaningful in that it was easy and convenient for using and helpful for self‐nutritional management of pregnant women with DM.

### AII‐5‐4

#### Outcomes after hospitalization of diabetes in older patients received influenza vaccination

**Y.‐C. Chou^1^, C.‐C. Liao^2^ and T.‐L. Chen^2^**

^1^Department of Physical Medicine and Rehabilitation, China Medical University Hospital^2^Department of Anesthesiology, Taipei Medical University Hospital

**Background**

The effects of influenza vaccination (IV) on diabetic outcomes remains unknown.

**Methods**

We used Taiwan's insurance claims data of 2008–2013 to conduct a study of 11976 patients ≥ 66 years receiving IV before diabetic admission within 1 year. Frequency matching was used to select 11976 non‐IV diabetic patients.

**Results**

Patients with IV had lower risk of 30‐day mortality (OR 0.75, 95% CI 0.61–0.93), decreased risk of postoperative intensive care, shorter length of hospital stay and lower medical expenditures after diabetic hospitalization compared with non‐IV patients.

**Conclusion**

**O**lder patients with IV showed better outcomes after diabetic hospitalization compared with non‐IV diabetic patients.

### AII‐5‐5

#### Severe hypoglycemia and risk of dementia in person with diabetes mellitus

**K. H. Ha^1,2^, J. Y. Jeon^1^, H. J. Kim^1,2^, K.-W. Lee^1^ and D. J. Kim^1,2^**

^1^Department of Endocrinology and Metabolism, Ajou University School of Medicine^2^Cardiovascular and Metabolic Disease Etiology Research Center, Ajou University School of Medicine

We investigated the association between severe hypoglycemia and risk of dementia in person with DM. A total of 67,485 people with DM include in the analysis. During a median of 10.5 years of follow‐up (interquartile range, 10.0–11.2), 8,951 people had a first diagnosis of dementia. Those who experienced a hypoglycemic event had a 2‐fold increased risk for developing dementia compared with those who did not have a hypoglycemic event (OR, 1.88; 95% CI, 1.65‐After adjusted confounding variables, the association between severe hypoglycemia and risk for dementia remained significant (OR, 1.18; 95% CI, 1.02–1.36). Our results provide evidence for an association between severe hypoglycemic events and dementia in person with diabetes.

### AII‐5‐6

#### Glycemic variability and in‐hospital mortality among elderly patients in the ICU

**A. A. Macalalad‐Josue^1,2,3^**

^1^Section of Endocrinology, Diabetes, and Metabolism, Department of Medicine, Philippine General Hospital^2^Department of Clinical Epidemiology, College of Medicine, University of the Philippines Manila^3^Department of Medicine, The Medical City

Glycemic variability (GV) affects the outcome of critically ill patients. To determine the effect of GV on inpatient mortality in the elderly, we did a retrospective cohort study with patients ≥65y/o admitted to ICU. GV indices were higher among those who died but there was no significant association. There was a trend towards greater inpatient mortality with increasing GV with mean glucose>180 mg/dL. Hence, GV is not a significant prognostic factor in critically elderly patients.

### AII‐6‐7

#### Association study of 14 single nucleotide polymorphisms and insulin resistance in a Chinese population

**Y. Huang, J. Yan, C. Hu and W. Jia**

Department of Endocrinology and Metabolism, Shanghai Jiao Tong University Affiliated Sixth People's Hospital

**Aims**

14 loci were selected to investigate the effect on predicting changes in insulin sensitive in a 9‐year prospective cohort in the Chinese population.

**Methods**

14 SNPs were selected and genotyped in a 9‐year prospective cohort (*n* = 1,179). Gutt‐ISI index and HOMA‐ISI index were used to evaluate insulin sensitive.

**Results**

rs2943641 (β = −7.316, *P* = 0.04) and rs3923113 (β = −5.986, *P* = 0.02) were significantly associated with changes in Gutt‐ISI index. Subjects carrying CC genotype of rs2943641, and those carrying GG genotype of rs3923113 were more insulin resistant assessed by Gutt‐ISI.

**Conclusions**

This study reports association of rs2943641 and rs3923113 with insulin resistance in a Chinese prospective cohort.

### AII‐6‐8

#### Role of Activin/FSTL3 in the glucose metabolism

**Y. OkazakiM^1^, N. Kobayashi^2^, A. Iwane^1^, T. Sasako^1^, H. Suwanai^1^, M. Kobayashi^1^, N. Kubota^1^, K. Hara^1^, T. Yamauchi^1^, K. Yoshimura^3^, I. Koshima^3^, H. Aburatani^4^, T. Kadowaki^1^ and K. Ueki^1,2^**

^1^Department of Diabetes and Metabolic Diseases, the University of Tokyo Hospital^2^Diabetes Research Center, Research Institute National Center for Global Health and Medicine^3^Plastic, Reconstructive and Aesthetic Surgery, the University of Tokyo Hospital^4^Research Center for Advanced Science and Technology, the University of Tokyo

We found that the expression of Follistatin like‐3 (FSTL3) in subcutaneous and visceral fat was increased at the very early stage of obesity and correlated with BMI.

The expression of FSTL3 was upregulated in fat samples of db/db mice. Overexpressing of FSTL3 exhibited impaired glucose metabolism and downregulation of FSTL3 improved insulin sensitivity.

FSTL3 is known to bind to Activin, and we found that expression level of G6Pase and PEPCK, key regulators of gluconeogenesis, was suppressed by treatment with recombinant Activin in primary hepatocyte.

We found that the effect of impaired glucose metabolism of FSTL3 appears to be though inhibition of Activin which exhibited lower glucose levels by suppression of gluconeogenesis.

### AII‐6‐9

#### Serum aryl‐hydrocarbon receptor transactivation assay reveals persistent nature of diabetogenic chemicals

**H. K. Lee^1^, J. T. Kim^1^, M. S. Oh^2^, H. S. Choi^3^, H. Nam Cho^4^, W. H. Park^5^ and Y. P. Kim^5^**

^1^Department of Internal Medicine, Eulji University^2^Department of Statostics, Ewha Women's University^3^Department of Internal Medicine, Kangwon National University Medical CollegeN^4^Department of Preventive Medicine, Ajou University Medical College^5^Department of Physiology, Kyunghee University College of Medicine

Serum AhR transactivation activity and mitochondrial inhibitor activity are predictive of future development of T2D among Koreans, suggesting that persistent organic pollutants is involved in the pathogenesis. Further analysis of Korean Genome Epidemiology study samples showed smokers had higher AHRTA (RR 12.14, 95%CI 2.766–53.13, *P* < 0.001) and ex‐smoker still had higher AHRTA (RR 3.2, 95%CI, 1.14–8.99, *P* < 0.027) than non‐smokers. Serum AHRTA and MIA reflected previous diabetic state; those subjects who had diabetes in 2002 had higher AHRTA and MIA and more diabetes had developed during 2002–2008 period among the subjects belonging to higher AHRTA and MIA quartiles. These observations suggest diabetogenic chemicals persist in human body.

### AII‐6‐10

#### Identifying a new pathway to regulate AMPK activity and autophagy under metabolic stress

**E. Yamada, S. Okada, R. Shibusawa, Y. Tagaya, A. Osaki, Y. Shimoda, T. Saito, T. Sato and M. Yamada**

Department of Medicine and Molecular Science

Metabolic stress is associated with dysfunctions of AMP‐dependent protein kinase (AMPK). Additionally pro‐inflammatory cytokines such as TNFα contributes to insulin resistance. Previously we reported that Fyn regulates AMPK activity by direct phosphorylation of Y426 of AMPKα. TNFα increased Fyn kinase activity and siRNA knockdown of Fyn prevented TNFα inhibition of AICAR stimulated AMPK activation. TNFα induced inhibition of autophagy was not observed when AMPKα was mutated on Y436. These data demonstrate that Fyn plays an important role of TNFα on autophagy via phosphorylation of AMPK.

### AII‐6‐11

#### Enhanced expression of Survivin plays distinct roles in adipocyte homeostasis

**L. Ju^1,2,3,4,5^, X. Zhang^6,7^, Y. Deng^8^, J. Han^1,2,3,4,5^, J. Yang^6,7^, S. Chen^1,2,3,4,5^, Q. Fang^1,2,3,4,5^, Y. Yang^1,2,3,4,5^ and W. Jia^1,2,3,4,5^**

^1^Shanghai Institute for Diabetes^2^Shanghai Key Laboratory of Diabetes^3^Shanghai Clinical Medical Centre of Diabetes^4^Shanghai Key Clinical Centre of Metabolic Diseases^5^Shanghai Jiao‐Tong University Affiliated Sixth People's Hospital^6^Institute of Endocrine and Metabolic Diseases^7^Ruijin Hospital, Shanghai Jiaotong University School of Medicine^8^The Affiliated Hospital of Qingdao University

Survivin is a gene that has been found to abundantly expressed in fetal tissues and the most common human tumors, and was considered absent in normal tissues in initial studies.

Here, we report that Survivin can be expressed upon insulin stimulation in vitro and under high fat diet feeding conditions in vivo. The ectopic expression of Survivin in 3T3‐L1 cells does not affect adipocyte differentiation, but rather inhibits isoproterenol‐stimulated adipocyte lipolysis via the β‐Adrenergic/cAMP pathway. Survivin also attenuates DNA damage‐related stress responses, as well as TNFα‐induced lipolysis. Taken together, the current findings suggest that Survivin may facilitate adipocyte maintenance in response to obesity‐induced adipocyte stress.

### AII‐6‐12

#### SFPQ, a pre‐mRNA splicing factor interacts with phosphorylated HELZ2 and plays a novel role in adipocyte differentiation

**A. T. Katano^1^, T. Satoh^1^, T. Tomaru^1^, S. Yoshino^1^, T. Takamizawa^1^, T. Watanabe^1^, T. Okamura^1^, S. Matsumoto^1^, K. Horiguchi^1^, Y. Nakajima^1^, S. Ishii^1^, A. Ozawa^1^, N. Shibusawa^1^, M. Mori^2^ and M. Yamada^1^**

^1^Department of Medicine and Molecular Science, Gunma University Graduate School of Medicine^2^Metabolic and Obese Research Institute

Understanding of molecular mechanism for adipocyte differentiation would contribute to the management of glucose intolerance, an obesity‐related disease. We previously identified HELZ2, a coregulator of PPARγ. To further elucidate the pathological process of obesity, we here isolated splicing factor proline/glutamine‐rich (SFPQ), a pre‐mRNA splicing factor as a protein that interacts specifically with tyrosine‐phosphorylated HELZ2. Knockdown of Sfpq in 3T3‐L1 cells resulted in reduced fat accumulation due to aberrant expression of transcription factors that play essential roles in the early stage of adipocyte differentiation and deranged pre‐mRNA alternative splicing. SFPQ may be a novel target for the treatment of obesity‐related disease.

### AII‐6‐13

#### Imeglimin increases insulin secretion in response to glucose as a unique mechanism of action depending on NAD synthesis

**S. Hallakou‐Bozec^1^, S. Bolze^1^, M. Kergoat^2^, P. Fouqueray^1^ and K. Takeda^1^**

^1^Poxel^2^Métabrain Research

Imeglimin (IME) is a novel glucose‐lowering agent improving insulin secretion in response to glucose (GSIS) and insulin sensitivity. IME is currently in phase 2b in Japan.

IME increases NAD content involving the NAD synthesis salvage pathway. Its GSIS effect does not involve classical insulin secretion effectors and is synergic with GLP‐1. In T2D patients IME increases insulin secretion.

IME increases b‐cell mass, decreases the number of apoptotic b‐cells and increases the proportion of proliferative b‐cells.

These results demonstrate that IME‐induced GSIS involves the NAD synthesis salvage pathway and may protect b‐cell mass.

### AII‐6‐14

#### Effect of liraglutide and monoclonal FGFR2‐IIIc antibody on the development of obesity and type 2 diabetes in mice

**K. Nonogaki**

Department of Diabetes Technology, Tohoku University Graduate School of Biomedical Engineering

We examined the effect of liraglutide, a glucagon‐likepeptide‐1 (GLP‐1) receptor agonist, and monoclonal fibroblast growth factor receptor 2c (FGFR2c) antibody on the development of obesity and type 2 diabetes in mice. Systemic administration of liraglutide significantly decreased food intake, body weight, blood glucose levels and the expression of FGFR2 in epididymal white adipose tissue at 24 h after its administration while having no significant effects on plasma insulin and glucagon levels in individually housed KKA^y^ mice. Treatment with FGFR2‐IIIc antibody significantly suppressed body weight gain and epididymal white adipose tissue weight by increased lipolysis without affecting food intake and blood glucose levels in KKA^y^ mice.

### AII‐6‐15

#### A limonene‐derivative purified from Sudachi peel upregulates sirt1 and improves lipid/glucose metabolism in HFD‐fed mice

**L. Miyamoto^1^, H. Aihara^1^, W. Xu^1^, M. Jin^1,2^, Y. Tomida^1^, T. Yamaoka^1^, N. Tanaka^2^, Y. Ikeda^3^, T. Tamaki^3^ and K. Tsuchiya^1^**

^1^Department of Medical Pharmacology, Institute of Biomedical Sciences, Graduate School of Tokushima University^2^Department of Pharmacognosy, Institute of Biomedical Sciences, Graduate School of Tokushima University^3^Department of Pharmacology, Institute of Biomedical Sciences, Graduate School of Tokushima University

Citrus Sudachi is a small sour typical seasoning in Japanese cuisine. A daily administration of Sudachi peel extended lifespan of ZDF rats. Here we determined active compounds of the sudachi peel. We successfully identified a limonene‐derivative as an active molecule by an index of TG‐reducing effects in cultured cells. It increased sirt1 expression levels, and the TG‐lowering effects was sensitive to nicotinamide, suggesting involvement of sirt1. In HFD‐fed mice, the compound improved glucose tolerance, fatty liver, serum TG and cholesterol levels with increase in sirt1 activities. Thus, it should be one of the active components of Sudachi peel regulating metabolism, which is supposed to be mediated by sirt1 upregulation.

## Poster Presentation Abstracts

### AI‐P‐1

#### Association between haptoglobin genotype and diabetic macroangiopathies in Chinese patients with type 2 diabetes

**S. Wang, R. Zhang, F. Jiang, C. Hu and W. Jia**

Shanghai Diabetes Institute, Shanghai Key Laboratory of Diabetes Mellitus, Shanghai Clinical Center for Diabetes, Shanghai Jiao Tong University Affiliated Sixth People's Hospital

Haptoglobin (Hp) is an acute‐phase protein that is crucial for the neutralization of oxidative damage. We aimed to investigate the relationship between polymorphisms in Hp (Hp1‐1, Hp2‐1 and Hp2‐2) and macroangiopathies in Chinese patients with type 2 diabetes (*n* = 6355). Hp genotyping was performed using TaqMan‐based real‐time PCR. Results showed that Hp^2^ allele was negatively correlated with carotid diameter (*P* = 0.0242), carotid intima‐media thickness (*P* = 0.0312) and Hp serum levels (*P* < 0.0001). After adjusting for variables, we found a significant association with diabetic macroangiopathies (OR = 0.859, 95%CI 0.745–0.990, *P* = 0.0354). Our data indicate that Hp polymorphisms were associated with diabetic macroangiopathies in a Chinese population.

### AI‐P‐2

#### GCK‐MODY is not rare in a Chinese community population

**Y. Ma^1^, X. Han^2^, L. Ji^2^, X. Zhou^2^ and Y. Li^1^**

^1^Beijing Pinggu Hospital^2^Peking University People's Hospital

The aim of our study is to estimate the prevalence of MODY2 in a Chinese community population. In a Chinese community population including 3345 subjects in Pinggu district, Beijing, China, 252 of 336 patients newly diagnosed with T2DM were screened for mutations in exons of GCK. Three known causative mutations (p.R43H, p.G44S, p.R377L) and two novel deleterious mutations (p.T82P, p.A259S) were identified in five patients, which didn't exist in 300 normal subjects. The proximate prevalence of MODY2 in the Chinese population ranged from 0.09% to 0.15%. Our study indicates MODY2 is not rare in Chinese population.

### AI‐P‐3

#### Predicting effects of G6PC2 on type 2 diabetes and impaired glucose regulation in a 9‐year prospective Chinese cohort

**F. Jiang^1,2,3^, C. Hu^1,2,3^ and W. Jia^1,2,3^**

^1^Shanghai Diabetes Institute, Shanghai Jiao Tong University Affiliated Sixth People's Hospital^2^Shanghai Key Laboratory of Diabetes Mellitus^3^Shanghai Clinical Center for Diabetes

The aim of this study was to investigate the predicting effects of common variants of G6PC2 on deterioration of blood glucose and change in glucose metabolism traits in in a community‐based 9‐year prospective cohort (*n* = 2,547) of Chinese population. Three SNPs (rs492594, rs2232328 and rs16856187) of G6PC2 were genotyped. Only rs16856187 has predicting effects on incidence of T2D and IGR with trend (*P* = 0.074) after adjusting for age, sex, BMI at baseline in logistic regression. Moreover, none of the three SNPs show predicting effects on changes in glucose metabolism traits. In this study we did not identify the predicting effects of G6PC2 variants on blood glucose deterioration and change in glucose metabolism traits.

### AI‐P‐4

#### The association of variations in COX1 and COX2 with type 2 diabetes and glucose metabolism in Chinese Han populations

**B. Xu^1,2,3^, C. Hu^1,2,3^ and W. Jia^1,2,3^**

^1^Department of Endocrinology, Shanghai Jiao Tong University Affiliated Sixth People's Hospital^2^Shanghai Diabetes Institute^3^Shanghai Key Laboratory of Diabetes Mellitus

**Aim**

Cyclooxygenases (COX) are vital enzymes in inflammation, proved to be associated with type 2 diabetes (T2D). We aimed to test whether polymorphisms in COX1 and COX2 are associated with T2D and glucose metabolism in Chinese.

**Methods**

We included 6822 individuals with OGTT data and genotyped tag SNPs of COX1 and COX2.

**Results**

rs3218625 of COX2 was correlated with T2D after adjusting for age, gender and BMI (OR = 0.74, 95% CI = 0.56 to 0.97, *P* = 0.031). Moreover, variants in both COX1 and COX2 displayed association with glucose related traits, including glucose level, insulin secretion and sensitivity (*P* = 0.002–0.049).

**Conclusion**

Our study suggests that COX might play a part in the prevalence of T2D and glycometabolism in Chinese Han populations.

### AI‐P‐5

#### Variations in KCNJ11 are associated with early‐onset type 2 diabetes mellitus in Chinese population

**L. Liu, L. Zhuang, X. Ge, J. Zhang, M. Li, R. Zhang, W. Zhao, Y. Chen, J. Yin, Y. Bao and W. Jia**

Department of Endocrinology & Metabolism, Shanghai Jiaotong University Affiliated Sixth People's Hospital, Shanghai Diabetes Institute, Shanghai Key Laboratory of Diabetes, 600 Yishan Road, Shanghai 200233, China

**Objectives**

To investigate if the E23K (G>A) or A190A (C>T) in KCNJ11 are associated with early‐onset T2DM in Chinese.

**Methods**

A case‐control study was conducted in 175 early‐onset T2DM patients who receive insulin (ins+, *n* = 57) or not (ins‐, *n* = 118), and 182 control subjects, who were genotyped by PCR‐sequencing.

**Results**

Higher E23K‐GA+AA and lower A190A‐TT in T2DM, especially in the ins+ group; higher 2 h plasma glucose and lower 2 h insulin in E23K‐AA (vs. E23K‐GG) of control group were observed (all *P* < 0.05). A190A‐TT had higher SBP than CC In ins+ group (*P* < 0.05).

**Conclusions**

The E23K may increase the risk of early‐onset T2DM which can be protected by A190A‐TT. The A190A‐TT or E23K‐GG may elevate the risk of hypertension in the Chinese.

### AI‐P‐6

#### Association of PAX4 polymorphism with diabetic retinopathy in Chinese population

**Y. Li, C. Hu and W. Jia**

Department of Endocrine, Shanghai Jiao Tong University Affiliated Sixth People's Hospital

**Objectives**

This study aims to investigate the association between rs10229583 near PAX4 and diabetic retinopathy (DR) susceptibility in a Chinese T2DM cohort.

**Methods**

We genotyped rs10229583 in T2D patients with DR, and without DR whose duration is over 5 years (*n* = 998 and 1,373, respectively). All patients underwent full ophthalmological examination.

**Results**

No evident significant association was found between DR and rs10229583 (*P* = 0.8031, OR = 1.021) in logistic analysis. Similar result was found (*P* = 0.7831, OR = 0.976) after adjusting for the diabetic duration, HbA1c, blood pressure and BMI.

**Conclusions**

Rs100229583 near PAX4 had no significant effects on DR in Chinese population.

### AI‐P‐7

#### Baseline level and change in serum albumin concentration and the risk of incident type 2 diabetes

**Y. C. Hwang^1^, W.‐J. Hong^2^, S.‐M. Jin^2^, J. C. Bae^2^, K. Y. Hur^2^, M.‐K. Lee^2^ and J. H. Kim^2^**

^1^Department of Medicine, Kyung Hee University School of Medicine^2^Department of Medicine, Sungkyunkwan University School of Medicine

Baseline serum albumin concentration was associated with an unfavorable metabolic phenotype and partly with future risk of type 2 diabetes. However, change in serum albumin could better explain the association between serum albumin concentration and diabetes risk than did a single measurement; that is, an increase in serum albumin concentration was inversely and independently associated with a lower risk for incident T2D.

### AI‐P‐8

#### Elevation in serum fibroblast growth factor 23 levels in normoglycemic first‐degree relatives of patients with diabetes

**X. Ma, X. Hu, Y. Luo, Y. Xu, Q. Xiong, X. Pan, Y. Bao and W. Jia**

Department of Endocrinology and Metabolism, Shanghai Jiao Tong University Affiliated Sixth People's Hospital; Shanghai Clinical Center for Diabetes, Shanghai Key Clinical Center for Metabolic Disease, Shanghai Diabetes Institute, Shanghai Key Laboratory o

The goal of this study was to explore alteration in serum fibroblast growth factor 23 (FGF23) levels and its value for identifying subclinical atherosclerosis in normoglycemic subjects with a first‐degree family history of diabetes (FHD). Serum FGF23 levels were higher in 312 subjects with a first‐degree FHD than in 1407 subjects without a FHD. A first‐degree FHD was positively and independently associated with serum FGF23 levels. In subjects with a first‐degree FHD, only those with serum FGF23 levels in the upper quartile were more likely to have an increased C‐IMT. As conclusion, the influence of a first‐degree FHD on serum FGF23 levels should be considered to avoid overestimating the cardiovascular disease risk.

### AI‐P‐9

#### Heavy alcohol drinking as a nonglycemic parameter to determine hemoglobin A1c in a Korean population

**D.‐J. Kim, J. Noh and J. Hong**

Department of Internal Medicine, Inje University Collegel of Medicine Ilsanpaik Hospital

Among the 24,594 participants who participated in the 2011–2013 Korea National Health and Nutrition Examination Survey (KNHANES), 12,923 participants were analyzed in this study. Logistic regression analyses showed that, when using the group that abstained as the control, the group that consumed alcohol ≥ 30 g/day was negatively associated with the risk of an HbA1c level of ≥5.7%, ≥6.1%, and ≥6.5% even after adjusting for confounding factors, including the FPG concentration, in this nationally representative sample of Korean adults. These results suggest that excessive drinking shifts the HbA1c level downward, which might complicate use of the HbA1c level for the diagnosis of diabetes or prediabetes.

### AI‐P‐10

#### Comparisons between early onset and late onset patients of the glucose control in newly diagnosed T2DM in China

**X. Cai, X. Gao, X. Han and L. Ji**

Peking University People's Hospital

To learn the baseline characteristics and the glucose control in early onset newly diagnosed type 2 diabetes patients, we performed an observational and prospective study in a cohort of newly diagnosed T2DM patients in China. A total of 5770 patients from 79 hospitals were included. The early onset patients were defined as patients with the diagnosed age no more than 40 years old. Totally 666 patients were early onset diabetes patients. Comparisons between early onset and late onset patients of the baseline characteristics indicated that in China, in newly diagnosed patients, early onset patients tended to be more males, more smokers, more obese, higher baseline HbA1c level, but decreased more after one year of treatment.

### AI‐P‐11

Withdrawn.

### AI‐P‐12

#### The association of CYP2C9 and CYP2J2 variants with type 2 diabetes risk in Chinese populations

**T. Wang^1,2^, R. Zhang^1,2^, C. Hu^1,2^ and W. Jia^1,2^**

^1^Shanghai Diabetes Institute,Shanghai Jiao Tong University Affiliated Sixth People's Hospital^2^Shanghai Key Laboratory of Diabetes Mellitus,Shanghai Clinical Center for Diabetes

CYP450‐mediated epoxyeicosatrienoic acids exert favorable effects on glucose homeostasis. We aim to test the impacts of CYP2C9 and CYP2J2 variants on type 2 diabetes (T2D) risk in Chinese populations. We totally genotyped 22 tagging loci of CYP2C9 and CYP2J2 in 6822 participants and collected the clinical measurements. We found that CYP2C9 rs1819173 was associated with T2D (OR = 1.19, 95%CI 1.10–1.28, *P* = 6.93 × 10^−6^) and it remained significant even adjusting for multiple testing (empirical *P* = 0.0003). Moreover, we observed that CYP2C9 rs1934967 and CYP2J2 rs10789082 and rs1593461 were associated with insulin secretion and sensitivity indies (*P* = 0.02–0.04). It is indicated that CYP2C9 and CYP2J2 may play an important role in glucose metabolism.

### AI‐P‐13

#### Retrospective review on the outpatients with type 1 diabetes mellitus at Chiba University Hospital

**J. Kumagai^1,2^, K. Ishikawa^1,2^, A. Kobayashi^1,2^, K. Kurita^1,2^, H. Inoue^1,2^, H. Yokoh^1,2^ and K. Yokote^1,2^**

^1^Clinical Cell Biology and Medicine, Graduate School of Medicine, Chiba University^2^Department of Diabetes, Metabolism and Endocrinology, Chiba University Hospital Chiba, Japan

Retrospective review of the medical records of outpatients with T1DM at Chiba University Hospital was performed in September 2016.

We enrolled 148 patients. The number of acute type, fulminant type, and slowly progressive insulin dependent diabetes mellitus (SPIDDM) were 104, 10, and 34 respectively. The age of onset of acute type was significantly younger than that of SPIDDM. The serum C‐peptide level and prevalence of retinopathy in SPIDDM were significantly higher than in other types.

The dose of bolus insulin in patients on pump one year after the induction of pump therapy was significantly lower than that of before the induction. The pump therapy, however, provided no improvements in HbA1c and indexes of blood glucose variability.

### AI‐P‐14

#### Effect of ethanolic leaf extract from Adenanthera pavonina (L) on blood sugar regulation in rats

**A. N. Sanjeewani^1,2^, D. B. M. Wickramarathne^3^ and P. H. P.Fernando^4^**

^1^Department of Pharmacy, Faculty of Allied Health Sciences, General Sir John Kotelawala Defence University^2^Postgraduate Institute of Agriculture, University of Peradeniya^3^Department of Pharmacy, Faculty of Allied Health Sciences, University of Peradeniya^4^Department of Biochemistry, Faculty of Medicine, University of Peradeniya

Objective was to investigate effect on BGC of ELEAP in rats. A group‐500 mg/kg/day of ELEAP and B group‐water. OGTT and starch tolerance test (STT) was done at day 10 and 17. Significant increase in BGC was observed in the A group. Shows hypoglycemic activity (HA) to control normal BGC. There is no anti‐HA,insulin like or releasing activity, or amylase activity.

### AI‐P‐15

#### Usage of CGM for management of glucocorticoid replacement therapy in adult patients with central hypoadrenalism

**A. Ozawa, T. Watanabe, T. Tomaru, S. Ishii, N. Shibusawa, S. Okada, T. Satoh and M. Yamada**

Department of Medicine and Molecular Science, Gunma University Graduate School of Medicine

Patients with adrenal insufficiency require glucocorticoid replacement. Some patients receiving sufficient doses of glucocorticoids occasionally exhibit morning headache, which may be caused by unrecognized hypoglycemia. CGM identified unrecognized hypoglycemia at midnight or early in the morning in 10 patients out of 11. Based on these findings, fine‐tune the dosing and regimens of glucocorticoid replacement were done. After the optimization, hypoglycemia disappeared and symptoms were improved in all patients. These findings demonstrated that CGM may represent a powerful tool for identifying hypoglycemia and optimizing therapies in patients with centarl hypoadrenalism.

### AI‐P‐16

Withdrawn.

### AI‐P‐17

#### Comparative study on prevalence and cause of hypoglycemic attacks observed at emergency room between 2010 and 2015

**K. Nemoto^1^, Y. Takayanagi^1^, Y. Maeda^1^, E. Tsuchiya^1^, T. Nishikawa^2^, M. Mimura^1^ and R. Moriwaki^3^**

^1^Department of Internal Medicine, Chiba Rosai Hospital^2^Department of Internal Medicine, Yokohama Rosai Hospital^3^Department of Emergency and Critical Care, Chiba Rosai Hospital

**Objective**

To compare the prevalence and cause of hypoglycemic attacks in diabetic patients visiting emergency room both year 2010 and 2015.

**Methods**

Diabetic patients with severe hypoglycemia in 2010 and 2015 were evaluated at emergency visit.

**Results**

Among the cases, 33 and 22 patients were detected severe hypoglycemia in 2010 and 2015. Most of severe hypoglycemic patients were attributed to either SU or insulin in both observed years.

Patients attributed to using SU significantly decreased from 77% to 36%. In contrast, patients attributed to insulin increased from 36% to 64%.

**Conclusion**

As DPP4 inhibitors have been widely used in Japan, severe hypoglycemic cases related to SU administration seems to have decreased in number.

### AI‐P‐18

#### Factors complicating diabetes management for foreign patients with diabetes in Japan

**M. Kishimoto, N. Ishimura, Y. Nagai and N. Tajima**

Department of Internal Medicine, Sanno Hospital

As the number of foreign visitors and residents in Japan increase, Japanese medical staff is increasingly likely to encounter foreign patients with diabetes. However, there are several factors complicating diabetes management for these patients. Diet therapy may be especially difficult to implement because eating habits are unique to each culture and ethnicity. Differences in language, cultural habits, healthcare insurance, and medications are other potential barriers. Based on our experiences, we analyzed these problems and concluded that to provide better diabetes care to foreign patients, efforts should be made to understand their cultural backgrounds, food habits, and the diabetes treatment guidelines used in their native countries.

### AI‐P‐19

#### Usefulness of glucose parameters obtained from Oral glucose tolerance test (OGTT) in Korean subjects

**T. S. Park^1,2^, H. Y. Jin^1,2^, H. S. Baek^1,2^ and K. A. Lee^1,2^**

^1^Department of Endocrinology and Metabolism, Chonbuk National University Medical School^2^Research Institute of Clinical Medicine, Chonbuk National University Hospital

We evaluated OGTT results from 494 subjects who had performed an OGTT. We measured the OGTT parameters (G0, G60, G120), insulin, C‐pep, HbA1c during OGTT. OGTT parameters significantly correlated with HbA1c, insulin, and C‐peptide in total subjects. OGTT parameters using the possibility of HbA1c ≥6.5% is the highest in G0 and G60 is the lowest. Using G0, G60 and G120, the highest rate of HbA1c ≥6.5% when G0 and G120 were used together, and the lowest when using G60 and G120. However, odds ratio of HbA1c ≥6.5% is the highest when G60 and G120 were used combined. From our results of OGTT, the useful result of diabetes diagnosis is G0 and G120, so the measurement and usefulness of G60 value in OGTT should be reviewed from large studies.

### AI‐P‐20

#### Serum 1,5‐anhydroglucitol levels slightly increase after a glucose load

**H. Su, X. Ma, J. Yin, Y. Wang, X. He, Y. Bao, J. Zhou and W. Jia**

Department of Endocrinology and Metabolism, Shanghai Jiao Tong University Affiliated Sixth People's Hospital, Shanghai Clinical Center for Diabetes, Shanghai Key Clinical Center for Metabolic Disease, Shanghai Diabetes Institute, Shanghai Key Laboratory o

The goal of this study was to explore short‐term changes in serum 1,5‐anhydroglucitol (1,5‐AG) levels after a glucose load. A total of 681 participants without a prior history of diabetes mellitus (DM) were enrolled. Serum 1,5‐AG levels were slightly elevated after a glucose load, the difference between peak and baseline levels (Δ1,5‐AG) was significantly lower in DM patients than in individuals without DM. Δ1,5‐AG was positively associated with baseline 1,5‐AG was also found. Although serum 1,5‐AG levels have been shown to decline with long‐term hyperglycemia, the present study showed that serum 1,5‐AG levels slightly increased after a glucose load.

### AI‐P‐21

#### Inverse association between fasting insulin levels and postprandial asprosin level changes in Type 2 diabetes patients

**S. Tokumoto, E. Okamura, M. Abe, S. Honjo and A. Hamasaki**

Centre for Diabetes and Endocrinology, Tazuke Kofukai Foundation, Medical Research Institute, Kitano Hospital

Asprosin (ASP) has recently been discovered as a glucogenic hormone secreted from adipose tissue. While ASP promotes hepatic glucose release, the pathophysiological role of ASP in patients with glucose intolerance remains unclear. We aimed to evaluate the relationship between ASP postprandial concentration kinetics and other biomarkers. 8 healthy subjects and 23 type 2 diabetes mellitus (T2DM) underwent a 2‐h meal tolerance test. In T2DM patients, postprandial reduction of ASP levels showed a significant negative association with fasting insulin levels (r = −0.41 *P* < 0.05). Fasting insulin levels were negatively correlated with postprandial ASP level reduction, suggesting that insulin could regulate postprandial ASP levels.

### AI‐P‐22

#### Response to 75g glucose tolerance tests: comparison of type 2 diabetes and healthy control

**S. Tserensodnom^1^, B. Byambatsogt^1^ and S. Sonomtseren^2^**

^1^Endomed Clinic^2^Mongolian National University of Medical Science

**Objective**

To compare response to 75 g‐OGGT in patients with newly diagnosed T2DM and healthy volunteers.

**Methods**

Each facility was enrolled 15 patients with newly diagnosed T2DM and 15 healthy controls, totally 30. OGGT wasconducted and samples were collected at 0 and 120 min after glucose administration.

**Results**

Response to OGGT was abnormal for all T2DM patients. Among healthy controls IFG were 4 (26.7%), IGT were 3 (20.0%) and abnormal HbA1C were 2 (13.3%).

**Conclusions**

Responses to 75 g glucose tolerance tests are abnormal for all T2DM patients andtwo third of their have a critical high level of blood glucose in last 3 months. Among healthy controls one third have an IFG and one fifth have an IGT.

### AI‐P‐23

#### HbA1C and mean glucose derived from continuous glucose monitoring assessment do not correlate in patients with HbA1c >8%

**Y. Tagaya, E. Yamada, Y. Nakajima, A. Osaki, Y. Shimoda, R. Shibusawa, T. Saito, T. Sato, S. Okada and M. Yamada**

Department of Medicine and Molecular Science Gunma University Graduate School of Medicine

**Aims**

Optimum therapy for patients with diabetes depends on both acute and long‐term changes in plasma glucose (PG) as assessed by HbA1c. We examined this relationship using continuous glucose monitoring (CGM).

**Methods**

51 patients (70% women, 30% male) were examined. HbA1c were compared with PG determined by CGM.

**Results**

Changes of HbA1c up to 8.0% showed a statistically strong relationship (R = 0.6713, *P* < 0.0001) with mean PG measured by CGM. However, when HbA1c was more than 8.0% there was no statistically significant relationship (R = 0.0498, *P* = 0.8298).

**Conclusions**

CGM data is a good clinical indicator of long‐term glucose control, however cautions in interpreting CGM data should be taken when the HbA1c are above 8.0%.

### AI‐P‐24

#### Comparison of markers between metabolically obese but normal weight and metabolically healthy but obese in Korean adults

**Y. H. Kang, A. R. Khang, H. W. Lee, D. W. Yi and S. M. Son**

Department of Internal Medicine, Pusan National University Yangsan Hospital, Pusan National University School of Medicine, Yangsan, Korea

The aim of this study was to compare multiple markers between metabolically obese but normal weight (MONW, *n* = 412) and metabolically healthy but obese (MHO, *n* = 518) in total 2,917 adults Vitamin D was significantly decreased in subjects with MONW (15.7 ± 7.1 ng/mL) compared with MHO (16.9 ± 6.4 ng/mL). However, fatty liver index (FLI) and cystatin C were significantly increased in subjects with MHO (FLI, 47.1 ± 21.1; cystatin C, 0.88 ± 0.16 mg/L) compared with MONW (FLI, 36.9 ± 20.8; cystatin C, 0.85 ± 0.16 mg/L). There were no significant differences in CRP, homocysteine, GGT, and HOMA‐IR between two groups. Low vitamin D may be more related with metabolically obese, but high FLI and cystatin C may be more related with obesity itself.

### AI‐P‐25

#### The association of TCF7L2 genetic variants with diabetic retinopathy in Chinese population

**X. Wang, R. Zhang, C. Hu and W. Jia**

Shanghai Diabetes Institute, Shanghai Jiao Tong University Affiliated Sixth People's Hospital

TCF7L2 has been confirmed to be associated with T2D and diabetic retinopathy (DR) in Caucasians. However, the effects of TCF7L2 variants on DR in Chinese is still unknown. We genotyped rs7903146 in T2D patients with and without DR (*n* = 998 and 1,373, respectively). No significant association was observed of rs7903146 and DR after adjusting duration, HbA1c, blood pressure and BMI in logistic analysis (OR = 0.869, P = 0.4011). In conclusion, TCF7L2 rs7903146 had no significant effects on DR in Chinese population.

### AI‐P‐26

#### One Stop Service for survey of diabetic complications

**C. S. Chen and P.‐S. Cheng**

Department of Endocriniology and Metabolism, Taipei City Hospital

**Introduction**

Diabetes mellitus with poor glycemic control was notorious for its chronic complications.

**Methods**

One visit in our clinics, we will provide survey of urine albumin excretion rate (UAE), non‐mydriatic retinal camera,and quantitative sensory testing (QST). Peripheral artery disease was also assessed by ankle brachial index (ABI). We will check the rate before and after the service.

**Results**

The percentage of four examinations were increased. Retinal examination from 67.6% to 72.8%, UAE from 63.5% to 79.8%, QST from 0% to 45%,and ABI from 89.9% to 95.1%.

**Conclusions**

One stop survey complications of diabetes were a patient‐center service. Integrated care of patient could increase the percentage of diagnosed complication.

### AI‐P‐27

#### Association between FTO gene polymorphisms and osteoporosis in patients with T2DM

**J. Guo^1^, W. Ren^2^, X. Li^1^ and G. Xi^2^**

^1^Department of Endocrinology, The second Affiliated Hospital of Shanxi Medical University^2^Department of Endocrinology, Shanxi Medical University, Shanxi Academy of Medical Sciences & Shanxi Dayi Hospital

**Background**

In this study, We investigated the association of FTO polymorphisms with BMD and other parameters in patients with T2DM.

**Methods**

120 patients were included in this study. The FTO polymorphisms rs1861868, rs1421085 and rs9939609 were determined. BMD Other clinical parameters were measured.

**Results**

Patients with osteoporosis were more likely to carry the rs9939609 A allele compared to normal patients with BMD. Two haplotypes (ACA and GCA) were associated with higher risk of osteoporosis. Patients without the ACA allele showed lower T‐score at the femoral neck and an increased risk of osteoporosis.

**Conclusion**

Genetic variations in FTO are associated with osteoporosis among patients with T2DM.

### AI‐P‐28

#### Risk of diabetes in patients of chronic obstructive pulmonary disease: a retrospective cohort study

**C.‐C. Liao and T.‐L. Chen**

School of Medicine, Taipei Medical University

We evaluated diabetes risk in chronic obstructive pulmonary disease (COPD) patients. We identified 14720 adults with COPD exacerbations (COPDe) and 29440 non‐COPD controls in 2000–2008. Diabetes events in 2000–2013 were identified in the follow‐up period. Adjusted HR of diabetes associated with COPD were calculated. The incidences of diabetes for patients with no COPD and COPDe were 2.4 and 5.2 per 1,000 person‐years, respectively. Patients with COPDe (HR 2.53) had increased risk of diabetes. The association between COPDe and diabetes risk was significant in both sexes and every age group. We concluded that COPDe was associated with diabetes risk.

### AI‐P‐29

#### Effect of sleep disorders in patients with diabetes mellitus

**S. Kubota, T. Kurose, S. Ueno, S. Okamoto, Y. Sakuramachi, K. Okamura, H. Kuwata, K. Watanabe, T. Hyo, N. Tanaka, Y. Hamamoto and Y. Seino**

Center for Diabetes, Endocrinology and Metabolism, Kansai Electric Power Hospital

We have attempted to evaluate the characteristics of sleep parameters and sleep quality of patients with diabetes mellitus using questionnaires on individual sleep quality. The participants asked the Pittsburgh Sleep Quality Index (PSQI). We analyzed 100 patients with diabetes mellitus. The average total score of PSQI was 4.39 ± 0.23. Patients with over 6 scores of PSQI are considered to have sleep disorder, and 32 patients out of 100 patients meet this definition and grouped as high PSQI. 68 patients were classified as low PSQI group. Systolic blood pressure in the group of the high PSQI was significantly higher than in low PSQI group. In addition average BMI of high PSQI was 23 ± 0, significantly lower than 26 ± 1 in the low PSQI group.

### AI‐P‐30

#### Long term risk of stroke in type 2 diabetic patients with diabetic ketoacidosis

**Y.‐L. Chen and K.‐J. Tien**

Division of Endocrinology and Metabolism, Department of Internal Medicine, Chi Mei Medical Center

We extracted claims data for 3572 type 2 diabetic patients with DKA and 7144 controls between 2000 and 2002. All patients were tracked until new stroke diagnosis, death, or end of 2011. Two hundred seventy of the 3572 patients with diabetic ketoacidosis and 404 of 7144 of the controls were diagnosed as having a new stroke, incidence rate ratio (IRR) 1.56 (95% CI 1.34–1.82; *P* < 0.0001). Patients with diabetic ketoacidosis had a higher risk of ischemic stroke than those without (IRR 1.62, 95% CI 1.34–1.96; *P* < 0.0001). After adjustment for patient characteristics and comorbidities, diabetic ketoacidosis patients were 1.55 times more likely to have a stroke than those without diabetic ketoacidosis (95% CI 1.332–1.813, *P* < 0.0001).

### AI‐P‐31

#### The impact of endothelial malfunction and postprandial hyperglycemia on adverse events among type 2 diabetic patients

**T. Sato^1^, Y. Azuma^2^, J. Kurimoto^2^, Y. Hodai^2^, T. Kinoshita^2^, E. Wada^2^, K. Yamaguchi^2^, T. Kato^2^ and A. Inagaki^2^**

^1^Division of Diabetes and Endocrinology, Masuko Memorial Hospital/Nagoya Daini Red Cross Hospital^2^Division of Diabetes and Endocrinology, Nagoya Daini Red Cross Hospital

Endothelial dysfunction associates with postprandial hyperglycemia. We conducted observational study of concecutive 108 type 2 diabetic patients and examined thereafter for 30 months the composite endpoint, including major adverse cardiovascular event. This cohort patients’ age, body mass index, HbA1c, eGFR, LDL cholesterol levels were 62.3 ± 13.6 years, 26.3 ± 4.9 kg/m2, 10.3 ± 2.0%, 69.9 ± 18.7 mL/min, 113 ± 36.2 mg/dL, respectively. Flow‐mediated dilatation (FMD) and 1‐h postprandial glucose levels (1hPPG) had negative correlation. Interestingly, eGFR, 1hPPG and FMD were all associated with the composite endpoint by logistic regression analysis. Postprandial hyperglycemia‐mediated endothelial malfunction may predict future adverse events.

### AI‐P‐32

#### The synergistic impact of apolipoprotein B/A‐1 and lipoprotein (a) on coronary artery calcification

**K. Park, J. Kim, S. Lee, J. You, J. Park, J. Nam, C. Ahn, K. Kim and Y. Kim**

Department of Internal Medicine, Gangnam Severance Hospital, Yonsei University College of Medicine

We investigated the influence of Apo B/Apo A‐1 and Lp(a) on coronary artery calcification (CAC) among healthy Korean adults. A total of 1081 participants were enrolled. Anthropometric profiles and Apo B, Apo A‐1, and Lp(a) were measured. Multi‐detector CT was used to measure coronary artery calcium score (CACS) and CACS>0 was defined as the presence of CAC. The prevalence of CAC significantly increased with Lp(a) and Apo B/Apo A‐1 levels. In the logistic regression analysis adjusted for multiple risk factors, odds ratio for the prevalence of CAC comparing the lowest Lp(a) and Apo B/Apo A‐1 group to the highest group was 2.554 (1.26–5.20) (*P* < 0.05). These results show that Apo B/Apo A‐1 and Lp(a) have a synergistic impact on prevalence of CAC.

### AI‐P‐33

#### Establishment of a zebrafish system for diabetes complications by novel acceleration plethysmogram and electrocardiogram

**T. Ito, M. Wakutsu, K. Ota, M. Koike, Y. Kondo, M. Sagawa, H. Sato, H. Takayasu, R. Tanaka, M. Nukuda, R. Marushita, N. Monma, J. Yamamoto, M. Omura and S. Yamazaki**

Department of Nutrition and Dietetics, Faculty of Family and Consumer Sciences, Kamakura Women's University

We identified 49 circulating miRNAs specifically regulated with insulin resistance, and two of which were predicted as miRNAs targeting arteriosclerosis‐related genes. So we developed an evaluating zebrafish system for arteriosclerosis and diabetic autonomic neuropathy by the acceleration plethysmogram and non‐aggression type electrocardiogram. We demonstrated that high fat diet significantly increased SDPTGAI showing arteriosclerosis, and caused patterns of electrocardiogram indicating coronary ischemia, and furthermore, significantly decreased parasympathetic nerve activity in power spectrum.

Zebrafish was shown to be a useful model to quickly evaluate arteriosclerosis, heart ischemia and early stages of diabetic autonomic neuropathy.

### AI‐P‐34

#### Fibroblast growth factor 23 strengthen the capacity of Framingham Risk Score to identify subclinical atherosclerosis

**X. Hu, X. Ma, Y. Luo, Y. Xu, Q. Xiong, X. Pan, Y. Bao and W. Jia**

Department of Endocrinology and Metabolism, Shanghai Jiao Tong University Affiliated Sixth People's Hospital, Shanghai Clinical Center for Diabetes, Shanghai Key Clinical Center for Metabolic Disease; Shanghai Diabetes Institute, Shanghai Key Laboratory o

The goal of this study was to investigate the relationship between serum fibroblast growth factor 23 (FGF23) levels and carotid intima‐media thickness (C‐IMT) in men with a low‐to‐moderate cardiovascular disease (CVD) risk assessed by Framingham Risk Score (FRS). This study enrolled 392 and 372 men with a low and moderate CVD risk, respectively. The results showed that men with high serum FGF23 levels, and no matter with a low or moderate CVD risk, were more likely to have an increased C‐IMT than those with low serum FGF23 levels and a low CVD risk. Serum FGF23 levels were independently and positively associated with C‐IMT. As a conclusion, serum FGF23 levels strengthen the capacity of the FRS for identifying subclinical atherosclerosis.

### AI‐P‐35

#### Low‐frequency and very low‐intensity ultrasound decreases blood pressure in hypertensive subjects with type 2 diabetes

**K. Nonogaki**

Department of Diabetes Technology, Tohoku University Graduate School of Biomedical Engineering

Despite lifestyle interventions and anti‐hypertension agents, hypertension remains difficult to control in some patients with type 2 diabetes. Here we show the effects of low‐frequency and low‐intensity ultrasound applied to the forearm on blood pressure (BP) and pulse rate in Japanese subjects with type 2 diabetes and hypertension. Systolic and diastolic BP, pulse rate, pulse pressure in the ultrasound treatment group were significantly lower than the baseline values in hypertensive subjects with type 2 diabetes, and lower than those of placebo controls. Low‐frequency and low‐intensity ultrasound irradiation to the forearm might have potential usefulness as a therapeutic application for clinic hypertension in subjects with type 2 diabetes.

### AI‐P‐36

#### Negative pressure wound therapy inhibit inflammation in diabetic patients with foot ulcerations

**R. He^1,2,3,4^, T. Wang^1^, F. Liu^1,2,3,4^ and W. Jia^1,2,3,4^**

^1^Department of Endocrinology and Metabolism, Shanghai Jiaotong University Affiliated Sixth People's Hospital^2^Shanghai Key Laboratory of Diabetes^3^Shanghai Institute for Diabetes^4^Shanghai Clinical Medical Centre of Diabetes

**Objective**

To evaluate the inflammatory signals involved in the effects of Negative pressure wound therapy (NPWT) on diabetic foot ulcers.

**Methods**

We enrolled 22 patients with diabetic foot ulceration, eleven treated with NPWT and others treated with traditional debridement. All the patients were treated and observed for 1 week. Granulation tissue harvested and analyzed using ELISA, Western blot and real‐time PCR.

**Results**

NPWT promote wound healing and significantly decreased the expression of TNF‐α, IL‐6 and iNOS. Western blotting and real‐time PCR indicated that NPWT decreased the level of IkB‐α and NF‐kB P65 and increased the level of ATF‐3 (all *P* < 0.05).

**Conclusion**

NPWT exerts an anti‐inflammatory effect in human diabetic foot wounds.

### AI‐P‐37

#### Adjuvant effects of diabetes on hypertensive neuropathy in spontaneously hypertensive rats

**H. Nukada^1,2^, R. Hotta^1^, M. Baba^3^, D. McMorran^2^, S. Ogasawara^4^ and S. Yagihashi^1,4^**

^1^The Nukada Institute for Medical & Biological Research^2^University of Otago^3^Aomori Prefectual Central Hospital^4^Hirosaki University

Hypertension causes neuropathy in spontaneous hypertensive rats (SHR) (Nukada, et al., Muscle Nerve 2016). We studied the potential for the additive effects of diabetes upon existing hypertension for the development of neuropathy. Neonatal STZ‐diabetes was induced in SHR and Wistar rats. SHR complicated with diabetes (SHR+DM), hypertension only (SHR), diabetes only (DM), and two controls; WKY and Wistar rats were studied. Nerve conduction velocity in SHR+DM was the slowest at age of 28 and 44 weeks. Morphological abnormalities, e.g. axonal atrophy of myelinated fibers, in SHR+DM was greater than in SHR or DM. These findings suggest that diabetes contributes to worsening of hypertensive neuropathy.

### AI‐P‐38

#### Factors associated with post discharge follow up among hospitalized patients with diabetic foot

**D. C. Bernardo, A. M. Lat and C. M. Sison‐Pena**

Section of Endocrinology, Diabetes and Metabolism, Department of Medicine, Philippine General Hospital

This study aims to identify clinical and demographic factors associated with post‐discharge follow up among patients admitted for one of the most common complications of diabetes, the diabetic foot.

Records of adults admitted for diabetic foot in the Philippine General Hospital from December 2011 to 2013 and subsequently discharged to the outpatient clinics were reviewed. Multiple logistic regression was done to derive associations between clinical, demographic variables and follow up.

Post‐discharge follow up rate is 22%. Among all other variables, non‐drinking status is associated with post‐discharge follow‐up (OR 1.98; CI 1.01 ‐ 3.88; *P* = .047). and may be a marker for good treatment‐seeking behavior in this population.

### AI‐P‐39

#### DPN Check, a nerve conduction testing device, is useful as the indicator for foot ulcer risk assessment

**M. Sumikawa^1^, T. Fujiwara^2^, K. Tatewaki^2^, N. Kawahara^2^, S. Saito^1^, H. Akasaka^3^, Y. Kuwamura^4^, Y. Sumikawa^5^ and M. Kubota^6^**

^1^Department of Nursing, School of Health Sciences, Sapporo Medical University^2^Sapporo Medical University Hospital^3^Department of Geriatric and General Medicine,Osaka University Graduate School of Medicine^4^Department of Nursing, Institute of Biomedical Sciences, Tokushima University Graduate School^5^Department of Dermatology, School of Medicine, Sapporo Medical University^6^Department of Sociology Kwansei Gakuin University, Health care center Kwansei Gakuin

**Aims/Methods**

NCV and NCA measurements of the sural nerve using DPN check were performed on diabetic patients to determine neuropathy severity and examine their relationships with foot ulcer risk assessment indicators.

**Results/Conclusions**

103 patients; age, 65.7 years; HbA1c, 7.3%.; NCV, 49.1 m/s (194 legs); NCA,9.8 μV (196 legs). Neuropathy severity was normal, mild, moderate, and severe in 112, 49, 32, and 10 legs, respectively. (1) NCV correlates with foot lesions, high HbA1c, diabetic complications, and smoking. (2) NCA correlates with diabetic complications and some vascular disorder indicators. (3) Severity of neuropathy was found to correlate with diabetic complications. Thus, DPN Check is useful in analyzing foot ulcer risk.

### AI‐P‐40

#### Healing progress of diabetic foot ulcer

**T. Boldbaatar^1^, O. Bavuu^2^ and S. Sonomtseren^2^**

^1^Endomed Clinic^2^Mongolian National University of Medical Science

**Objective**

The aim of this study was to evaluate healing progress of DFU.

**Methods**

Cohort study included T2DM patients with foot ulcer visited Endomed diabetic foot clinic, Mongolia last two years.

**Results**

Mean age of DM was 59.4 ± 9.56 years old and mean diabetes duration was 11.44 ± 6.4 years and mean HbA1C was 10.09 ± 1.96%. DFU healed er with foot amputation was 5 patients (AKA 2, BKA 1, digits 2). Recurrence of DFU was 72% (once 48%, multiple 24%). Primary healed DFU <1 month was 60%, 1–3 months were 20%, 3–6 months were 12%, non healed more 6 months 4%, and mortality was 4%.

**Conclusions**

Two third DFUs were primary healed <1 month, one fifth within 1–3 months, one tenth within 3–6 months and one fifth healed with amputation.

### AI‐P‐41

#### Screening for diabetic autonomic neuropathy using a blood pressure/pulse wave measuring apparatus

**N. Kato, M. Kato and H. Toshima**

Kato Clinic of Internal Medicine

We developed a simple test of autonomic function in diabetes.

**Materials and Methods**

In 77 patients with diabetes and 22 healthy volunteers, the cardio‐ankle vascular index (CAVI, an index of peripheral vessel stiffness) was measured on a blood pressure/pulse wave apparatus (Vasera 1500, Fukuda Denshi). The standard deviation /mean ratio for the height of 5 consecutive pressure pulse waves (CVWH) was used to assess sympathetic function.

**Results**

CAVI was significantly higher and CVWH was significantly lower in the diabetes group (both *P* = 0.0001). In patients on oral agents, CVWH was positively correlated with vibratory sensation and negatively correlated with CAVI.

**Summary**

CVWH could be a screening test for diabetic autonomic neuropathy.

### AI‐P‐42

#### Comparison of uric acid levels and mortality and risk of renal‐replacement therapy in DM and non‐DM CKD patients

**C.‐Y. Wu^1^, C.‐L. Lee^2,3^, M.‐J. Wu^1^, K.‐H. Shu^1^, C.‐H. Chen^1^, D.‐M. Yu^1^, Y.‐W. Chung^1^, S.‐T. Huang^1^, S.‐F. Tsai^1^ and Y.‐C. Lo^1^**

^1^Division of Nephrology, Department of Medicine, Taichung Veterans General Hospital, Taiwan^2^Division of Endocrinology and Metabolism, Department of Medicine, Taichung Veterans General Hospital, Taiwan^3^Department of Medical Research, Taichung Veterans General Hospital, Taiwan

Hyperuricemia is associated with CVD, mortality, renal outcome and the metabolic syndrome. However, the cut‐off value is unknown in DM and non‐DM CKD patients.

We enrolled 1776 DM patients and 2605 non DM patients with CKD stage 3, 4, 5 from 2007 to 2014. The end‐point was all cause mortality and receipt of renal‐replacement therapy (RRT).

Results suggest that in patients with DM and CKD stage 3, 4, 5, the risk of all cause mortality is not increased when serum uric acid levels are <8.0 mg/dl. However, in patients without DM, all cause mortality is not increased when serum uric acid levels are <8.0 mg/dl. The risk of RRT was associated with increased serum uric acid levels. However, no difference in risks of RRT was noted in the two groups.

### AI‐P‐43

#### Hemorheological approach for screening diabetic nephropathy in type 2 diabetes

**J. Kim, K. Park, J. Yu, S. Lee, J. Nam, J. Park, C. Ahn, K. Kim and Y. Kim**

Department of Internal Medicine, Gangnam Severance Hospital, Seoul, Korea

**Background**

We measured various hemorheologic parameters in type 2 diabetes patients and assessed their possible role as a diagnostic tool for diabetic nephropathy.

**Methods**

Hemorheologic parameters, including erythrocyte deformability, elongation index (EI), critical shear stress (CSS), and aggregation index (AI) were measured.

**Results**

There were significant differences in Elongation index at 3 Pascal (EI at 3 Pa) in CKD and diabetic nephropathy. Fibrinogen/EI at 3 Pa suggesting 860 mg/dL% as a cut off point for diabetic nephropathy with the sensitivity of 74% and specificity of 62%.

**Conclusions**

Fibrinogen/EI is a sensitive parameter measured via point‐of‐care testing for screening diabetic nephropathy in patients with type 2 diabetes.

### AI‐P‐44

#### Arg913Gln of SLC12A3 gene promotes development and progress of DN‐ESRD in Chinese type 2 diabetes mellitus

**L. Liu, L. Zhuang, X. Ge, J. Zhang, M. Li, R. Zhang, W. Zhao, Y. Chen, J. Yin, Y. Bao and W. Jia**

Department of Endocrinology & Metabolism, Shanghai Jiaotong University Affiliated Sixth People's Hospital, Shanghai Diabetes Institute, Shanghai Key Laboratory of Diabetes, 600 Yishan Road, Shanghai 200233, China

**Objective**

To evaluate the association of the SLC12A3‐Arg913Gln variation with ESRD risk in Chinese T2DM patients.

**Methods**

T2DM patients (*n* = 372) with diabetic retinopathy were classified into the non‐DN control group (*n* = 151, duration of diabetes >15 years) and the DN‐ESRD group (*n* = 221; ESRD due to T2DM, receiving hemodialysis), who were genotyped by PCR‐sequencing.

**Results**

Higher GA+AA genotype in DN‐ESRD subjects was observed compared to control group (*P* < 0.01); GA+AA carriers had higher UAER and DBP than GG in control subjects (both *P* < 0.05).

**Conclusions**

The SLC12A3‐Arg913Gln variation may predict the development and progression of DN‐ESRD in Chinese patients with T2DM undergoing hemodialysis via elevated blood pressure and UAER.

### AI‐P‐45

#### Aberrant STRA6 and CRBP1 signal pathway is involved in development of diabetic nephropathy via mitochondrial dysfunction

**S. S. Jang^1,2^ and C. C. Hung^1,2^**

^1^Department of Internal Medicine, Kaohsiung Medical University Hospital^2^Graduate Institute of Medicine, Kaohsiung Medical University

Reduction of retinoids, CRBP1 in db/db mice kidney was found. Mitochondrial dysfunction ian initiator of DKD. We hypothesized aberrant STRA6 and CRBP1 contribute to DKD via mitochondrial dysfunction. We found that binding activity of RBP4 with STRA6, CRBP1, retinol, RA, RARα, and mitochondrial function (MnSOD, cytochrome c, ATP synthase, mitochondrial potential) decreased but caspase 3 and collagen 1 increased in DM kidney and HG‐cultured HK‐2 cells. CRBP1 transfection recovered STRA6 signal and renal injury in HG‐cultured HK2 cells. ROS inhibitor and MnSOD transfection rescued aberrant STRA6 signal and renal injury. This study indicates that aberrant STRA6 and CRBP1signaling involve the process of DKD via mitochondrial dysfunction.

### AI‐P‐46

#### The association of a genetic variant in SCAF8‐CNKSR3 with diabetic microvascular disease in a Chinese population

**L. Jin, C. Hu and W. Jia**

Shanghai Diabetes Institute

Genomewide association studies found rs955333 was associated with DKD in EA, AA and MA. We aimed to investigate the association between the variant rs955333 with DKD susceptibility in 1884 Chinese T2D patients. Associations of the variant rs955333 with DKD as well as DR susceptibility and related quantitative traits were evaluated. The variant rs955333 was not associated with DKD in our samples, while subjects with genotype GG were associated with DR (*P* = 0.047, OR = 0.5525[0.308, 0.9911]), and it also showed association with AER (*P* = 0.024, Beta = −0.1812[−0.339, −0.024]). Our data suggests the variant rs955333 was not associated with DKD but showed association with DR in the Chinese T2D patients.

### AI‐P‐47

#### Abnormal thermal thresholds of lower extremities is significantly associated with low TBI in type 2 diabetic patients

**Y.‐J. Sheen^1,2^, W. H.‐H. Sheu^3,4,5,6^, J.‐L. Lin^1^, C.‐T. Bau^1^ and C.‐D. Kao^7^**

^1^Division of Endocrinology and Metabolism, Department of Internal Medicine, Taichung Hospital, Ministry of Health and Welfare^2^Department of Public Health, China Medical University^3^Division of Endocrinology and Metabolism, Department of Internal Medicine, Taichung Veterans General Hospital^4^School of Medicine, National Defense Medical Center^5^School of Medicine, National Yang‐Ming University^6^Institute of Medical Technology, National Chung‐Hsing University, Taichung^7^Division of Neurology, Department of Internal Medicine, Taichung Hospital, Ministry of Health and Welfare

We enrolled 725 type 2 diabetic patients with quantitative sensory test for thermal thresholds and ankle‐brachial index/ toe‐brachial index (TBI) examinations. Excluding patients with a history of apparent cardiovascular disease, arrhythmia, end‐stage renal disease, malignancy, and diagnosed peripheral neuropathy. A total of 539 study subjects had impaired thermal thresholds. After adjusting for gender and age, abnormal thermal thresholds was significantly associated with low TBI and low eGFR (*P* < 0.05). Fasting plasma glucose and HbA1C remain independently associated with impaired thermal thresholds after adjusting for confounding factors. Abnormal sensory test of lower limbs is associated with peripheral artery disease in type 2 diabetes.

### AII‐P‐1

#### Analysis of the Syndecan‐4 gene expression control mechanism in MIN6 cell

**I. Takahashi, K. Yanase, S. Hatakeyama, T. Mizokami and K. Nata**

Department of Medical Biochemistry, School of Pharmacy, Iwate Medical University

Heparan sulfate (HS) proteoglycans (PGs) comprise a core protein to which extracellular sugar chains are attached. We recently found that HS core protein Syndecan‐4 (Sdc4) are necessary for maintaining insulin secretory mechanism. So analysis of the regulation of Sdc4 expression is important for better understanding diabetes. We analyzed the sequence, methylation in Sdc4 promoter, and executed the deletion reporter assay using Sdc4 expressing and non‐expressing mouse pancreatic β‐cell line, MIN6 cells. Sdc4 promoter sequences showed no change in both cells, while specific methylation on the promoter was detected in Sdc4 non‐expressing cells only. Furthermore, deletion analysis revealed two important promoter regions for Sdc4 expression.

### AII‐P‐2

#### Differentiation of human induced pluripotent stem cells into insulin‐producing cells

**M. J. Kim, Y.‐H. You, J.‐W. Kim and K.‐H. Yoon**

Department of Endocrinology, The Catholic University of Korea

We have developed a highly efficient step‐wise protocol to direct insulin‐producing cell from diabetes patient‐specific iPS cell lines. For differentiation into insulin producing cells, we divided into 4 stages. We regulated the glucose concentration and used several small molecules in each steps and adenovirus for Pdx‐1 overexpression in the stabilizing stage. In final step, we reaggregated the cells like an islet cluster. Interestingly, aggregated cells showed expression of insulin gene and insulin positive cells. To functional test, we performed glucose‐stimulated insulin secretion. Interestingly, insulin secretion was a statistically significantly increased by high glucose treatment in differentiated cells.

### AII‐P‐3

#### Generation of the beta‐like cells differentiated into the Insulin‐producing cells from Mouse small intestine.

**S. H. Lee, J.‐W. Kim and K.‐H. Yoon**

Department of Endocrinology and metabolism, The Catholic University of Korea

Aim of our study was to generate the insulin‐producing cells form small intestine using an adenoviruses (Pdx‐1, MafA, Beta2/NeuroD, (PMB)). Adenoviruses delivered from duodenum to colon in whole intestine, and identified infection with the Ad‐Pdx1 and triple Ad‐PMB respectively in mouse small intestine. At 4 weeks after injection, insulin mRNA expressed in small intestines. In additionally, insulin+ cells detected in a part of crypts and villus located of small intestine tissues by immunostaining. These data indicated that Pdx‐1, MafA, Beta2/NeuroD facilitate the differentiation into insulin producing cells of mouse intestinal cells. In conclusion, small intestine is an accessible and abundant source of insulin‐producing cells.

### AII‐P‐4

#### Chronic arsenic exposure impairs pancreatic β‐cell function

**C. Carmean M^1^, A. Kirkley G^2^, N. Yokoi^1^, R. Hoshikawa^1^, K. Sugawara^1^, R. S. M^2,3^ and S. Seino^1^**

^1^Division of Molecular and Metabolic Medicine, Kobe University^2^Division of Biological Sciences, University of Chicago^3^Section of Endocrinology, Diabetes, and Metabolism, Department of Medicine, University of Chicago

Clinical cross‐sectional studies have reported a correlation between chronic arsenic exposure and the prevalence of type 2 diabetes mellitus. To investigate the molecular underpinnings of these observations, a mouse model of chronic arsenic exposure was developed. We observed glucose intolerance and impaired β‐cell response to hyperglycemia in mice following 8 weeks of exposure to inorganic arsenic in drinking water. Chronic exposure (3 days) to physiological concentrations of arsenic decreased glucose‐stimulated insulin secretion in the MIN6‐K8 β‐cell line, with no effects observed during acute (30 min) exposure. These studies raise the plausibility that arsenic exerts its diabetogenic effects through β‐cells.

### AII‐P‐5

#### Small molecules exert anti‐apoptotic effect on ICAs

**B. Chandravanshi and R. Bhonde**

School of Regenerative Medicine, Manipal University

Transplantation of pancreatic islets is the most reliable treatment for Type 1 diabetes. However cell death mediated by hypoxia is considered as one of the main difficulties hindering success in islet transplantation. The aim of our experiment was to investigate the role of small molecules in survival of Islet like cell aggregates (ICAs) engineered from umbilical cord matrix under oxygen deprived condition (<5%O2). ICAs were analyzed for cell death via fluorosceindiacetate/propidium iodide (FDA/PI) staining, estimation of Caspase 3 and free radical release in presence and absence of small molecules. The samples were also analysed for the presence of hypoxia inducible factor 1α (HIF1α) at both transcriptional and translational level. The addition of small molecules showed profounddefensive effect on ICAs under hypoxicenvironment as evidenced by their viability and insulin secretion compared to untreated ICAs. The combinations of Eicosapentaenoic acid (EPA), Docosahexaenoicacid (DHA) and metformin and EPA,DHAandγ amino butyric acid (GABA) acted as anti‐apoptotic agentsforhuman ICAs when exposed to 1% O2 for 48 h. The combinations of the small molecules reduced the total reactive oxygen species and malonaldehyde (MDA) levels and enhanced the production of glutathione peroxidise (GPx) enzyme under hypoxic conditions. Finally the increase in HIF1α at both protein and gene level confirmed the defensive effect of the additives in hypoxia. These results suggest that the combination of small molecules maintained the viability and functionality of the ICAs in hypoxia by up‐regulating HIF1α expression and down regulating the Caspase 3 activity.

### AII‐P‐6

#### Whole exome sequencing identifies a novel INS mutation as a hot spot mutation of maturity‐onset diabetes of the young

**C. Hu^1,2,3^ and W. Jia^1,2,3^**

^1^Shanghai Diabetes Institute, Shanghai Jiao Tong University Affiliated Sixth People's Hospital^2^Shanghai Key Laboratory of Diabetes Mellitus^3^Shanghai Clinical Center for Diabetes

We aimed to discover novel mutations leading to special types of diabetes which are misdiagnosed as type 2 diabetes in the Chinese population, and explore potential molecular mechanisms. We performed whole‐exome sequencing in 31 ‘T2D’ patients and found a novel mutation p.Ala2Thr in INS gene as a hot spot mutation of MODY. This variant was observed in 0.09% of cases in the replication study. Functional studies in INS1‐E cells showed that this mutation impaired beta cell function through inducing endoplasmic reticulum stress.

### AII‐P‐7

#### ATP and Ca^2+^ dynamics in pancreatic b‐cells: ex vivo analysis using ATP biosensor GO‐ATeam1

**R. Usui, D. Yabe, M. Fauzi, S. Tokumoto, H. Tatsuoka, Y. Tahara, M. Ogura, K. Nagashima and N. Inagaki**

Department of Diabetes, Endocrinology and Nutrition, Graduate School of Medicine Kyoto University, Kyoto, Japan

ATP dynamics in b‐cells was evaluated by ATP biosensor GO‐ATeam1. The dissociated islet cells were subjected to fluorescent microscopy to evaluate ATP and Ca^2+^ dynamics simultaneously during GIIS. The whole islets were subjected to two‐photon excitation microscopy to evaluate ATP dynamics in individual b‐cells. ATP increased by 1.2‐fold as early as ˜60s after glucose was increased; with Ca^2+^ elevation ˜80s after the ratio increase. In the whole islets, the average ratio increased by 1.1‐fold when glucose was increased. The ratio was increased in ˜30% of the cells examined. ATP rise proceed Ca^2+^ elevation in b‐cells during GIIS. Intracellular ATP did not elevate in some b‐cells, suggesting heterogeneity of b‐cells’ response to glucose.

### AII‐P‐8

#### GPR120 mediated pdx1 signaling protects against palmitic acid‐induced pancreatic beta‐cell dysfunction

**Y. Wang and P. S. Leung**

School of Biomedical sciences, The Chinese University of HongKong

As an unsaturated FFA sensor,GPR120 action in beta cells remains unexplored. Our results showed that GPR120 was expressed in beta cells and its stimulation with DHA or GSK restored palmitic acid (PA) downregulated the expression of insulin and pdx1 in MIN6 and islets. But the pdx1 level could not be rescued within GPR120 knockdown cells. In vivo,GPR120 KO mice displayed hyperglycemia and insulin resistance. We found that the pdx1 expression was decreased and the effects of GPR120 activation were not seen in GPR120 KO mouse islets compared with WT islets. The data suggests that GPR120 is protective against PA‐induced beta‐cell dysfunction via the mediation of pdx1.

### AII‐P‐9

#### Sphingosine kinase 1‐interacting protein (SKIP) is a novel regulator of glucose‐stimulated insulin secretion

**Y. Wang, S.‐i. Harashima, Y. Liu, R. Usui and N. Inagaki**

Department of Diabetes, Endocrinology and Nutrition, Graduate School of Medicine, Kyoto University, 606‐8507, Japan

Sphingosine kinase 1‐interacting protein (SKIP) is highly expressed in pancreatic b‐cells but not a‐cells. Compared to wild type (SKIP^+/+^) and their islets, (1) intraperitoneal glucose tolerance test showed a decrease in blood glucose levels and an increase in insulin levels, but no enhancement by exendin‐4 (Ex‐4) in SKIP^−/−^ mice; (2) glucose stimulated insulin secretion (GSIS) was amplified more with no enhanced effect by Ex‐4 in SKIP^−/−^ islets. (3) ATP and cAMP content were not altered between the islets; and (4) depolarization‐evoked, PKA and cAMP‐mediated insulin secretion were not affected in SKIP^−/−^ islets. These results indicate that SKIP modulates GSIS and influences glucose sensitivity in a KATP channel‐ and cAMP‐independent manner.

### AII‐P‐10

#### Association of Akt activation and adiponectin‐enhanced glucose stimulated insulin secretion in beta‐cells*

**H. Zhu^1^, X. Li^1^, S. Wang^1^, C. Wang^1,2^ and W. Jia^1,2^**

^1^Shanghai Diabetes Institute, Shanghai Jiao Tong University^2^Department of Endocrinology and Metabolism, Shanghai Jiao Tong University Affiliated Sixth People's Hospital

The aim of the study was to explore the mechanisms on adiponectin‐enhanced GSIS. INS‐1 cells were transfected with recombinant adenoviruses encoding APPL1, or its shRNA. Exposure of INS‐1 (832/13) cells to full‐adiponectin at 16.7 mmol/L glucose increased insulin secretion. It was abolished when the cells were pretreated with high levels of glucose/ palmate for 48 h, or APPL1 shRNA. Furthermore, this effect was associated with an inhibition of ATP production. Similar to IGF‐1, addition of adiponectin in INS‐1 (832/13) cells increased p‐Akt and p‐Erk expression and knocking down of APPL1 abolished the phosphorylation of Erk, but not Akt. These findings indicate a role of Akt activation in adiponectin‐enhanced GSIS in pancreatic beta‐cells.

### AII‐P‐11

#### Protection against high‐fat diet induced obesity in Helz2‐deficient mice by enhancing hepatic leptin sensitivity

**S. Yoshino^1^, T. Satoh^1^, T. Tomaru^1^, A. Katano‐Toki^1^, K. Horiguchi^1^, S. Matsumoto^1^, Y. Nakajima^1^, S. Ishii^1^, A. Ozawa^1^, N. Shibusawa^1^, S. Okada^1^, M. Mori^2^ and M. Yamada^1^**

^1^Department of Medicine and Molecular Science, Gunma University Graduate School of Medicine^2^Metabolic and Obese Research Institute

Obesity arises from impairment of energy homeostasis, which is centrally coordinated by leptin by activating the long form of leptin receptor (Leprb). We here show that mice fed a high‐fat diet (HD) and patients with hepatosteatosis exhibit upexpression of hepatic Helz2, which functions as a transcriptional coregulator for nuclear receptors including peroxisome proliferator‐activated receptorγ in vitro. To explore physiological importance of Helz2, we generated Helz2‐deficient mice and analyzed their metabolic phenotypes. Helz2‐deficient mice showing hyperleptinemia associated with central leptin resistance are protected against HD‐induced obesity and significantly upregulates hepatic Leprb expression.

### AII‐P‐12

#### Association of circulating miR‐29 with non‐alcoholic fatty liver disease

**Z. He, R. Zhang, C. Hu and P. J. Wei**

Department of Endocrinology and Metabolism, Shanghai Jiao Tong University affiliated Sixth Poeple's Hospital

The aim of this study was to examine the association of miR‐29 with NAFLD. Totally 276 individuals were enrolled in this study, including 67 individuals with both NAFLD and T2D, 73 individuals with NAFLD but no T2D, 68 individuals with T2D but no NAFLD and 68 controls. Logistic analysis was used to evaluate the association of miR‐29a‐c and NAFLD. It showed that miR‐191 was the most stably expressed reference miRNA. miR‐29a expression level was showed to be associated with NAFLD in individuals without T2D (OR = 5.960, *P* = 0.0304) but not in individuals with T2D (OR = 1.824, *P* = 0.5003). While meta‐analysis showed that miR‐29a was associated with NAFLD (OR = 1.239, *P* = 0.041). In this study, we showed that miR‐29a was associated with NAFLD.

### AII‐P‐13

#### Depletion of sphingosine kinase 1‐interacting protein (SKIP) induces metabolically healthy obesity

**Y. Liu, S.‐i. Harashima, Y. Wang and N. Inagaki**

Department of Diabetes, Endocrinology and Nutrition, Graduate School of Medicine, Kyoto University

SKIP is expressed in pancreatic b‐cells and intestinal K‐ and L‐cells. We examine the role of SKIP in incretin secretion and metabolic changes under normal diet conditions. SKIP‐mCherry knock‐in (SKIP^−/−^) mice with or without GIP (SKIP^−/−^/GIP^−/−^) were generated. In SKIP^−/−^, body weight and fat composition were gained; fatty liver was not induced; TG and T‐Cho levels were significantly decreased; pro‐inflammatory makers in liver were not increased; insulin, GIP and GLP‐1 secretion were promoted during OGTT. SKIP^−/−^/GIP^−/−^ eliminated the phenotype of SKIP^−/−^ except a decrease in TG and T‐Cho levels. In conclusion, depletion of SKIP induces metabolically healthy obesity by regulating insulin and incretin secretions and lipid metabolism.

### AII‐P‐14

#### The Drosophila RNF34 modulates lipid metabolism and exercise ability

**P. Wei1, C. Hu^1^, W.‐P. Jia^1^ and J. Wang^2^**

^1^Department of Endocrinology and Metabolism, Shanghai Jiao Tong University Affiliated Sixth People's Hospital, Shanghai Diabetes Institute, Shanghai Clinical Center for Diabetes, Shanghai, China^2^Department of Anatomy, Histology and Embryology, Neuroscience Division, Shanghai Jiao Tong University School of Medicine, Shanghai, China

We have reported that RNF34 is a bona fide E3 ubiquitin ligase for PGC‐1α and negatively regulates brown fat cell metabolism, but whether RNF34 plays a role as an E3 ligase for PGC1‐α in muscle is still unknown. Here, we show that tissue‐specific knockdown of the Drosophila RNF34 in muscle increases mitochondrial DNA content, the mRNA levels of the dPGC‐1 target genes and the negative geotaxis ability, reduces Drosophila dilp3 mRNA level and counteracts high‐fat‐diet induced high abdominal triglyceride amount. Knockdown of dPGC‐1 suppresses the dRNF34 knockdown phenotypes, which suggests the effects of dRNF34 are mediated through dPGC‐1. Our results might provide a new therapeutic approach for treatment of diabetes.

### AII‐P‐15

#### Analysis of age‐dependent leptin‐signaling in the leptin receptor deficient mice

**P. P. Wijang^1^, M. Ushikai^1^, E. Arimura^1,2^, M. Abe^1^, H. Kawaguchi^1^ and M. Horiuchi^1^**

^1^Department of Hygiene and Health Promotion Medicine, Graduate School of Medical and Dental Sciences, Kagoshima University^2^Department of Life and Environmental Science, Kagoshima Prefectural College, Kagoshima, Japan

Leptin is known to regulate energy homeostasis and food intake regulation in adult phase, but this function is scarcely investigated in neonatal period. We examined the age‐dependent leptin‐signaling dynamics in leptin receptor deficiency (db) mice. The body weight changes of db mice were occurring in adulthood. The impairment of db mice thermogenesis has shown in neonatal period and adulthood. The hypothalamus feeding neuron gene expression in neonatal db mice showed similar to wild type of mice. Leptin receptor is fully functional for energy homeostasis and food intake regulation in adulhood. However, the feeding regulation of leptin signaling shows age‐dependent manner,in contrast to energy homeostasis regulation.

### AII‐P‐16

#### Follistatin‐like 1 protects cardiomyoblasts from injury induced by hyperglycemia

**W. Chen, G. Xu and Z. Shen**

Institute for Cardiovascular Science of Soochow University

Diabetic cardiomyopathy is the leading cause contributing to morbidity and mortality in diabetic patients. We and others have demonstrated cardioprotection of follistatin‐like 1 (FSTL1) under excessive nitric oxide, myocardial infarction, and pressure overload. Here, we report that high glucose markedly elevates FSTL1 expression in H9c2 cells. Furthermore, FSTL1 treatment significantly attenuates hyperglycemia‐induced cardiac cell death. Our study provides novel insights into the therapeutic potential of FSTL1 in treatment of diabetic cardiomyopathy.

### AII‐P‐17

#### Anti‐diabetic effects of erythritol and associated molecular mechanisms investigated at ex vivo and type 2 diabetic rats

**M. Shahidul Islam, C. I. Chukwuma and R. Mopuri**

Department of Biochemistry, University of KwaZulu‐Natal (Westville Campus)

The study investigated the anti‐diabetic effects of erythritol and possible mechanisms at ex vivo and type 2 diabetic rats. Ex vivo results revealed that erythritol dose‐dependently enhanced muscle glucose uptake with or without insulin. Results from type 2 diabetic rats showed that erythritol delayed gastric emptying and reduced small intestinal glucose absorption as well as postprandial blood glucose rise, especially in diabetic animals. Additionally, erythritol improved glucose tolerance, insulin secretion, muscle/liver hexokinase and liver glucose‐6 phospahtase activities and muscle Glut‐4 and IRS‐1 mRNA expression in diabetic animals. Hence, erythritol may be a useful dietary supplement for managing hyperglycemia, particularly for T2D.

### AII‐P‐18

#### Senescent CD8^+^ T cells is functionally activated in subjects with prediabetes

**H.‐S. Yi^1^, J. T. Kim^2^, J. M. Mim^1^, K. H. Joung^1^, J. H. Lee^1^, K. S. Kim^1^, H. J. Kim^1^, M. Shong^1^ and B. J. Ku^1^**

^1^Department of Internal Medicine, Chungnam National University School of Medicine^2^Department of Medical Science, Chungnam National University School of Medicine

Chronic inflammation is associated with metabolic diseases. However, functional characteristics of T cells in patients with prediabetes are not understood. Gene expression profiling of peripheral blood mononuclear cells from normal controls and drug‐naïve patients with diabetes was undertaken using microarray analysis. Gene annotation enrichment analysis showed that most of the upregulated genes in the diabetes group are implicated in T cell activation and the inflammatory response. Pro‐inflammatory cytokine and cytotoxic granule proteins were also highly expressed in senescent CD8^+^ T cells from patients with prediabetes. This study suggests a causal link between T cell senescence and prediabetes in humans.

### AII‐P‐19

#### T cells analysis of oral combined antibiotic treatment in STZ‐induced diabetes mice model

**Z.‐c. Liu^1^, Y. Song^1^, Z.‐y. Zhao^2^ and Q.‐s. Hu^2^**

^1^The Diabetes Research Institute of The Shijiazhuang Second Hospital^2^The Shijiazhuang Second Hospital

**Objective**

To investigate the relation between immune homeostasis and the development of diabetic symptoms in Type 1 diabetes model.

**Methods**

One group of C57 mice received combined antibiotics treatment for 4 weeks before STZ injection, and than were injected with STZ. Another group of mice received only STZ injection. T cells were test after 72 h when the whole STZ‐injection process was finished.

**Results**

The cumulative diabetes incidence was significantly lower for the combined antibiotic group. The percentage of Treg cells and CD4 + T cells in mesenteric lymph nodes was higher than the control group.

**Conclusion**

These results indicate that oral combined antibiotic influencd the diabetes incidence in STZ‐induced diabetes model.

### AII‐P‐20

#### Apps ameliorates diabetic nephropathy progression by transcriptional repression of TGFβ1 through interaction with Nrf2

**P. Gao and J. Liu**

Department of Endocrine, Shanghai No.6 Hospital Affiliated to Shanghai Jiaotong University, Shanghai, China

TGFβ1 is a well‐distinguished mediator of progressive renal fibrosis in diabetic nephropathy (DN). Herein, we reported that Apps was a critical upstream regulator of TGFβ1 expression in the early stage of DN. The increased expression of Apps was negatively correlated with TGFβ1 in AAV‐renal vein‐injected/STZ induced mice. However, the expression of Apps decreased in the late stage of DN, when the expression of TGFβ1 was highly upregulated and the renal fibrosis got worsen. Apps synergistically interacted with Nrf2 in a time‐ and dose‐dependent manner. After activation by Nrf2, Apps was recruited into the nuclear and bound to the promoter of TGFβ1. Our findings may provide novel clues for the treatment of DN in early stage.

### AII‐P‐21

#### Perilipin 1 in macrophage protects the progression of atherosclerosis via the change of macrophage polarity

**K. Yamamoto, H. Miyoshi, K. Y. Cho, A. Nakamura and T. Atsumi**

Division of Rheumatology, Endocrinology and Nephrology at the Graduate School of Medicine, Hokkaido University

Perilipin 1 (PLIN1) is expressed in macrophages, presumambly playing a role in the development of atherosclerosis. We assessed the effect of PLIN1 overexpression. PLIN1 transgenic mice (Tg) were crossed with apolipoprotein E knockout mice (KO). C57BL/6J, KO and Tg/KO received a normal chow for 20 weeks. Aorta were collected at the end of the study for quantification of atheroma lesions and histology. Atherosclerotic lesions and M1 macrophage content increased in KO. PLIN1 in macrophages helps protect against atheroma progression via the change of macrophage polarity.

### AII‐P‐22

#### Short‐ and long‐term (6‐ and 24‐month) effects of exenatide in patients with type 2 diabetes

**O. Ebisui^1^, K. Ohno^1^, H. Tokunaga^1^, K. Akesaka^1^, T. Ueda^1^, M. Tamaki^1^ and Y. Hara^2^**

^1^Department of Diabetes and Endocrinology, Ehime Prefectural Central Hospital^2^Department of Diabetes, Ehime Prefectural Imabari Hospital

**Purpose**

We evaluated the effects of exenatide on HbA1c for 6 and 24 months in type 2 diabetics in our hospital.

**Methods**

1. Subjects: 97 patients (42 males, 55 females). 2. Before the administration of exenatide, HbA1c were evaluated every month for two years.

**Results**

The mean age, and HbA1c were 57.5 ± 12.5 years, and 9.7 ± 1.4%, respectively. <After 6 months> (1) The mean HbA1c was 8.1 ± 1.5%. (2) A 1% or greater decrease in HbA1c was achieved in 60 (61.8%). <After 24 months> (1) The mean HbA1c was 8.42 ± 1.6%. (2) A 1% or greater decrease in HbA1c was achieved in 48 (49.5%).

**Conclusion**

Based on these results, exnatides significantly improved glycemic control in about half of type 2 diabetic patients for 6‐and 24‐month.

### AII‐P‐23

#### Retrospective analysis between the efficacy of combination therapy of liraglutide and basal insulin and β‐cell function

**Y. Sakuramachi^1^, D. Yabe^2^, Y. Hamamoto^1^, T. Kurose^1^ and Y. Seino^1^**

^1^Center for Diabetes, Endocrinology, and Metabolism, Kansai Electric Power Hospital, Kyoto, Japan^2^Department of diabetes, Endocrinology and Nutrition, Graduate School of Medicine, Kyoto University, Kyoto, Japan

Twenty‐four patients who changed from intensive insulin therapy to combination therapy of liraglutide and basal insulin were retrospectively analyzed to identify associations of C‐peptide index (CPI) and SUIT index with achievement of HbA1c < 7.0% at 54 week. When 6 patients achieved HbA1c target was compared with 18 patients did not, CPI and SUIT index were not significantly different (CPI: 0.93 ± 0.27 vs. 1.11 ± 0.15, *P* = 0.57; SUIT: 37.2 ± 10.5 vs. 38.4 ± 4.9, *P* = 0.92). ΔHbA1c of 54 weeks and CPI or SUIT index did not show significant correlation. In conclusion, HbA1c‐lowering effect of combination therapy of liraglutide and basal insulin when switched from intensive insulin therapy is suggested to be independent of remaining β‐cell function.

### AII‐P‐24

#### Outcomes of efficacy and safety with alogliptin in eGFR study: results of 156 weeks follow up

**T. Hyo^1^, T. Kurose^1^, H. Kuwata^1^, K. Watanabe^1^, N. Tanaka^1^, Y. Hamamoto^1^, A. Kuroe^2^, T. Iwakura^3^, N. Takahashi^4^, H. Suzuki^5^, K. Oida^6^, N. Kitano^7^, A. Kanamori^8^, A. Kubota^9^, K. Yasuda^10^, H. Yokoyama^11^ and Y. Seino^1^**

^1^Center for Diabetes, Endocrinology and Metabolism, Kansai Electric Power Hospital^2^Division of Diabetes and Metabolism, Department of Internal medicine, Hikone Municipal Hospital^3^Division of Endocrinology and Metabolism, Department of Internal medicine, Kobe City Medical Center General Hospital^4^Takahashi Family Clinic^5^Koharu‐naika^6^Fukui Chuo Clinic^7^Division of Diabetes and Endocrinology, Department of Internal medicine, Hyogo Prefectural Amagasaki General Medical Center^8^Kanamori Diabetes Clinic^9^Kubota Clinic^10^Department of Diabetology and Endocrinology, Saiseikai Noe Hospital^11^Jiyugaoka Medical Clinic, Internal Medicine

**Objective**

Alogliptin is a selective inhibitor of dipeptidyl peptidase 4 (DPP‐4) titrated with renal function level. In order to evaluate the efficacy and safety of alogliptin based on renal function in T2DM, we designed prospective observational study (eGFR study).

**Results**

Study participants were all Japanese and 724 male and 373 female. Mean age was 63.5 ± 12.0 years, HbA1c was 7.6 ± 1.1%, fasting plasma glucose was 144.3 ± 35.0 mg/dl, eGFR was 74.8 ± 21.7 mL/min./1.73 m^2^, body weight was 65.8 ± 13.8 kg, the dosage of alogliptin was 22.2 ± 5.7 mg.

After 156 weeks follow up HbA1c was 7.1 ± 0.8% (*P* < 0.01), fasting plasma glucose was 134.6 ± 38.3 mg/dl (ns), eGFR was 68.8 ± 20.1 mL/min./1.73 m^2^ (*P* < 0.01), and body weight was 62.5 ± 12.0 kg (*P* < 0.01).

### AII‐P‐25

#### Efficacy and safety of evoglipitin monotherapy in patients with type 2 diabetes

**D.‐M. Kim^1^, S. Park^2^, M. Lee^2^, K. Yoon^3^ and J. Park^1^**

^1^Department of Internal Medicine, Hallym University College of Medicine^2^Department of Internal Medicine, Sungkyunkwan University School of Medicine^3^Department of Internal Medicine, College of Medicine, The Catholic University

**Background & Aims**

To evaluate the efficacy and safety of the new DPP‐4 inhibitor evogliptin in drug‐naive patients with type 2 diabetes.

**Materials and methods**

In this study, 160 patients were randomized to evogliptin 5 mg or placebo for 24 weeks. The primary outcome was mean changes from baseline in HbA1c.

**Results**

The mean baseline HbA1c levels of both group was 7.2%, and evogliptin provided significant placebo‐corrected reductions in HbA1c from baseline of ‐0.23% at 24 weeks. The response rate (HbA1c <6.5%) was significantly higher in the evoglipitin group than placebo.

**Conclusion**

In this 24‐weeks study, evoglipitin monotherapy significantly improved glycemic control and was well tolerated in patients with type 2 diabetes.

### AII‐P‐26

#### Identical hypoglyceamic effect of teneligliptin with linagliptin switched from sitagliptin in type 2 diabetes with CKD

**Y. Wada^1^, Y. Hamamoto^2^, S. Honjo^1^, E. Okamura^1^, M. Abe^1^, J. Fujikawa^3^ and A. Hamasaki^1^**

^1^Center for Diabetes and Endocrinology, Tazuke Kofukai Foundation Medical Research Institute Kitano Hospital^2^Division of Nutrition and Metabolism, Yutaka Seino Distinguished Center for Diabetes Research, Kansai Electric Power Hospital^3^Laboratory Medicine, Tazuke Kofukai Foundation Medical Research Institute Kitano Hospital

**Background**

We investigated to compare the effectiveness between teneligliptin and linagliptin in Japanese patients with CKD.

**Methods**

A total of 28 outpatients (mean age:72.1 ± 10.9 SD years, BMI:24.6 ± 3.6 kg/m^2^, serum creatinine: 1.5 ± 0.7 mg/dl, HbA1c:7.5 ± 1.3%) who were administered 20 mg of teneligliptin (*n* = 13; male9) or 5 mg of linagliptin (*n* = 15; male10) switched from 50 mg of sitagliptin were retrospectively extracted. Only those who with eGFR less than 60 mL/min/1.73 m^2^ at baseline were selected.

**Results**

HbA1c levels, body weight and serum creatinine levels were not changed in both groups.

**Conclusion**

The hypoglyceamic effect of teneligliptin was identical to linagliptin in Japanese patients with chronic kidney disease during 6 months.

### AII‐P‐27

#### Dipeptidyl peptidase‐4 inhibitor‐induced bullous pemphigoid: report of three cases

**S. Yoshiji, T. Murakami, S.‐i. Harashima, R. Ko, R. Kashima, D. Yabe, M. Ogura, K. Nagashima and N. Inagaki**

Department of Diabetes, Endocrinology and Nutrition, Kyoto University Graduate School of Medicine, Kyoto, Japan

**Background**

Bullous pemphigoid (BP) is a severe adverse event induced by dipeptidyl peptidase‐4 (DPP‐4) inhibitors. Here we report BP in the elderly treated with the agent.

**Case presentation**

Two men at the age of 86 and 81 and a woman at the age of 83 developed generalized pruritic skin blisters. Two men were treated with lingaliptin for 9 and 15 months, respectively; a woman with linagliptin for 15 months switching to sitagliptin for additional 10 months. BP was diagnosed histologically. Antibodies to BP180‐NC16A were detected in all the cases.

**Conclusion**

It took longer time to develop BP in our cases. BP should be noted no matter if older people with type 2 diabetes are safely treated with DPP‐4 inhibitors over time.

### AII‐P‐28

#### SGLT2 inhibitor and DPP4 inhibitor co‐administration in Thai diabetic patients

**N. Yenseung, S. Jirarungsaritkul, S. Sarakom, Y. Thewjitcharoen, E. Wanothayaroj, S. Krittiyawong, S. Nakasatien and T. Himathongkam**

Diabetes and Thyroid Center, Theptarin Hospital

Because of its unique mechanism of action, the use of SGLT2 inhibitors in patient treated with DPP4i may provide synergistic effect. This retrospective study used data between Nov 2014 and Jun 2016. A total of 156 patients who continue to use at least 6 months (baseline A1C 8.8 ± 1.5%) were included in analysis. Among DPP4i‐treated patients, overall mean A1C reduction when compared with non DPP4i‐treated patients was 0.9 ± 1.3% and 0.4 ± 1.2%, respectively. The proportion of DPP4i‐treated patients achieving HbA1c <7% was only 27.3%. While the combination of the two drug classes are being increasingly incorporated into clinical practice, careful patients selection accompanied by appropriate realistic treatment goal counseling should be done.

### AII‐P‐29

#### The relationship between morning spot urinary glucose parametersand HbA1c in patients with T2D after taking SGLT2i

**B. W. Lee, S. Kim, Y. Lee, E. Kang and B. Cha**

Yonsei University

At baseline, subjects showed wide range of HbA1c (7.55% to 9.05%) levels. SGLT2i therapies showed a significant decrease in HbA1c (from 8.1% to 7.5%; *P* < 0.001), as well as increase in spot urinary glucose concentration (22.0 mg/dl to 3859.0 mg/dl; *P* < 0.001) and spot UGCR (urinary glucose creatinine ratio) (0.19 to 47.5; *P* < 0.001). Change of HbA1c was positively correlated with that of spot UGCR (R = 0.177; *P* < 0.098). After multivariate adjustment, greater increase of UGCR were associated with delta HbA1c (after minus before HbA1c) (standardized beta = 0.269; *P* = 0.007) and eGFR CKD‐EPI (standardized beta = 0.501; *P* = 0.001).

This suggests urinary glucose excretion is not a good indicator of therapeutic response to SGLT2i in T2DM.

### AII‐P‐30

#### Type2 Diabetes complicating with NASH can be effectively controlled by Laennec^®^ through normalizing Iron Metabolism

**Y. Hamada**

Hamada Clinic for Gastroenterology

Recently it was elucidated that hepcidin can express in pancreatic βcells, which means that the pancreas could contribute to both glucose and iron metabolism. In type 2 diabetes complicating with NASH, remarkable declines of serum ferritin and HbA1c were observed after treating with Laennec^®^ (derived from human placenta). We divided 62 NASH cases (all liver biopsied) into two groups. By infusing Laennec^®^, serum ferritin level declined from 274.2 ± 310.4 ng/mL (before)to 58.3 ± 42.7 (after) (*P* < 0.01). Serum ALT and HbA1c level declined significantly. The improvement of type 2 diabetes complicating with NASH by Laennec^®^ suggests the importance of iron regulation on type 2 diabetes which shows of hyperferritinemia.

### AII‐P‐31

#### Clinical characteristics according to the use of metformin in Korean patients with type 2 diabetes mellitus

**I. G. Ha^1^, S. Y. Rhee^1^, S. W. Han^1^, S. K. Cho^1^, S. Chon^1^, S. H. Kim^2^, K. J. Ahn^1^, S. H. Baik^3^, Y. Park^4^, M. S. Nam^5^, K. W. Lee^6^ and J. T. Woo^1^**

^1^Department of Endocrinology and Metabolism, Kyung Hee University School of Medicine^2^Division of Endocrinology and Metabolism, Department of Medicine, Cheil General Hospital and Women's Healthcare Center, College of Medicine, Dankook University, Seoul, Korea^3^Division of Endocrinology and Metabolism, Department of Internal Medicine, College of Medicine,Korea University, Seoul, Korea^4^Department of Internal Medicine, College of Medicine, Hanyang University, Guri, Korea^5^Department of Internal Medicine, College of Medicine, Inha University, Incheon, Korea^6^Department of Endocrinology and Metabolism, College of Medicine, Ajou University, Suwon, Korea

This study was designed to identify the clinical characteristics and determinants of metformin use in clinical practice in Korean T2DM patients by using the baseline data of prospective, multicenter clinical registry.

Metformin users had significantly higher glycated hemoglobin, waist and hip circumference than non‐users. Lipid profile, creatinine, malignancy, depression and microvascular complication such as retinopathy, neuropathy, overt proteinuria were significantly lower in metformin users. But there was no difference in the prevalence of macrovascular complication.

**Conclusion**

There were significant differences in clinical characteristics and prevalence of comorbidities according to the use of metformin in Korean T2DM patients.

### AII‐P‐32

#### The effectiveness of Continuous Glucose Monitoring (CGM) in self‐management of people with diabetes

**M. Gushiken^1^ and Y. Omlor^2^**

^1^Department of Global health, Ryukyu University School of Medicine^2^University of the Ryukyus Hospital, Okinawa, Japan

The aim of this study was to assess the effectiveness of CGM in self‐management of diabetes.

The Problem Areas in Diabetes (PAID) survey was conducted for twenty patients before and after the use of CGM. The student't‐test were used to analyze associartion between the PAID score and the eight factors:gender, academic backgrund, having job or not, diabetic type, duration of diabetes, treatment, diabetic complications, and educational history in diabetes. The total PAID score was 25.6 ± 16.2 before and 21.8 ± 14.9 after using CGM (n.s.). HbA1c (NGSP) was 8.2 ± 2.2 (%) before and 7.8 ± 1.4 (%) after (n.s.). PAID score showed a significant association with patients having jobs in reducing the emotional burden due to the treatment (*P* < 0.05).

### AII‐P‐33

#### Evaluation psychometric properties of patients type 2 diabetes

**B. Munkhtur^1,2^, E. Yanjmaa^1,2^ and E. Yanjmaa^2^**

^1^Mongolian National University of Medical Sciences^2^School of Nursing, Mongolian National University of Medical Sciences

Examine psychometric properties of a Mongolian version of the PAID scale in patients with Type 2 diabetes in Mongolia. This study subjects were who visited the Diabetes centers. Cross‐sectional survey was participants with type 2 diabetes patients and included only the ones who met inclusion criteria and agreed with informed consent. At the baseline study were PAID Questionnaire in educated group 45 ± 17.4 at the 3 months 43.1 ± 14.1, and 6 months decreased 42.3 ± 13.6. Problem Areas in Diabetes Mongolian version were significantly different during the in non‐educated diabetic patients.

### AII‐P‐34

#### Happiness among patients with diabetes is affected by diabetes control and support

**F. Mano^1^, K. Ikeda^1^, Y. Uchida^2^, I.‐T. L. Huai‐Ching^2^, E. Joo^1^, M. Okura^3^ and N. Inagaki^1^**

^1^Department of Diabetes, Endocrinology and Nutrition, Graduate School of Medicine, Kyoto University^2^Kokoro Research Center, Kyoto University^3^Kyoto University Hospital

We investigated the impact of diabetes on happiness in an interdependent culture, Japan. We did a survey on 241 outpatients with diabetes using questionnaires and analyzed the data from 193 patients with type 2 diabetes. HbA1c had significant correlations with diabetes self‐care (r = −.17, *P* = 0.02), diabetes support (r = −.24, *P* < 0.01), happiness (r = −.24, *P* < .01) and age (r = −.23, *P* < .01). When divided by median age, multiple regression analysis showed that HbA1c (β =−.23, *P* = .02), diabetes support (β = .23, *P* = .02) and perceived limitations (β = −.31, *P* = .01) were significant independent predictors for happiness in the younger group. Intervention focusing on diabetes support and perceived limitation might be the next step to improve diabetes care.

### AII‐P‐35

#### Impact of individual and spontaneous goal setting in life for patients with diabetes mellitus

**S. Ueno^1^, N. Tanaka^1,2,3^, H. Higashiyama^2^, Y. Hamamoto^1,3^, T. Kurose^1,3^ and Y. Seino^1,3^**

^1^Centor for Diabetes, Endocrinology and Metabolisum Kansai Electric Power Hospital^2^Division of Medical Education, Kansai Electric Power Medical Research Institute^3^Yutaka Seino Distinguished Center for Diabetes Research, Kansai Electric Power Medical Research Institute

To investigate what content of declaration as long‐term goal in life leads favorable effect on the subsequent glycemic control and self‐management behavior.

Patients who participated in Diabetes Educational Program (*n* = 283, HbA1c: 9.6 ± 2.0%). They were categorized into five groups according to their goals in life: ‘to live a long life (group A)’, ‘to improve their health (B)’, ‘to enjoy their job or hobby (C)’, ‘to dedicate to others (D)’ and no comments (E).

HbA1c levels at 12 month were significantly lower in C+D than E (*P* < 0.05). Drug reduction rate in 12 months was significantly higher in C+D (*P* = 0.0353).

Individual and spontaneous goal settings were associated with better glycemic control with less medical therapy.

### AII‐P‐36

#### The glycemic control and behavior change after decrease of diabetic education frequency

**Y.‐C. Lai^1,2^, T.‐J. Chang^1^, Y.‐D. Jiang^1^, Y.‐H. Chen^1^, C.‐S. Wang^3^, Y.‐J. Wang^3^, S.‐F. Huang^3^, H.‐Y. Peng^4^, H.‐J. Chen^4^ and L.‐M. Chuang^1,2,5^**

^1^Department of Internal Medicine, National Taiwan University Hospital^2^Graduate Institute of Clinical Medicine, National Taiwan University^3^Department of Nursing, National Taiwan University Hospital^4^Department of Dietetics, National Taiwan University Hospital^5^Institute of Preventive Medicine, College of Public Health, National Taiwan University

This study population enrolled diabetes who received diabetic education more than 2 years. 91 received non‐intensified education and 322 received intensified education. At follow‐up, the effect of education frequency on HbA1c was insignificant over 3 years. However, the percentage of HbA1c<7% was higher in the group of non‐intensified education than intensified education at the 1‐year and 2‐year follow‐up. Drug adherence was better in the group of non‐intensified education as compare to group of intensified education at 2‐year follow‐up. We conclude that decrease of diabetic education frequency did not show impact on HbA1c and drug adherence in patient who had trained for coping with diabetes.

### AII‐P‐37

#### Nurses’ implementation and opinion of assessment of oral health behavior in patients with diabetes

**Y. Kuwamura^1^, M. Sumikawa^2^, E. Sakamoto^3^, I. Takikawa^4^, H. Yamato^4^, H. Uemura^5^, S. Kishida^1^, T. Nagata^3^ and M. Matsuhisa^6^**

^1^Department of Nursing, Institute of Biomedical Sciences, Tokushima University Graduate School^2^Department of Nursing, School of Health Sciences, Sapporo Medical University^3^Department of Periodontology and Endodontology, Institute of Biomedical Sciences, Tokushima University Graduate School^4^Tokushima University Hospital^5^Department of Preventive Medicine, Institute of Biomedical Sciences, Tokushima University Graduate School^6^Diabetes Therapeutics and Research Center, Tokushima University

**Aims**

To describe (1) nurses’ implementation of assessment of diabetes oral health behavior tool in patients with diabetes and (2) their opinion on the items of DiOHAT©, and discuss how to help the patients carry out oral health behavior.

**Methods**

Self‐written questionnaire.

**Results**

Response rate 48%;15 female nurses; mean age 46 yr. The average rate of implementation of all items was 44% and individual factors were perceptions 56%, status 49%, behavior 39%, and information 37%. The proportion counting the patient's total number of teeth was 40%.

**Conclusion**

The implications of assessing oral self‐care and counting the number of teeth showed low rates. More research is needed to motivate nurses to carry out oral assessments.

### AII‐P‐38

#### Characteristics and efficacy of our diabetes support team: a physician's viewpoint

**Y. Kouchi^1^, K. Hirayama^1^, T. Takenaka^1^, Y. Sakiyama^1^, R. Kushiro^1^, D. Kira^1^, A. Nakamura^1^, S. Sakai^1^, H. Kuramoto^1^ and Y. Takagi^2^**

^1^Diabetes Support Team, Orimoto Hospital^2^Nephrology, Orimoto Hospital

**Introduction**

Treatment of diabetes mellitus is based on team approach. We describe the characteristics and efficacy of our DST.

**Methods**

The monthly numbers of patients with diabetes of changing in HbA1c were analyzed.

**Results**

There were 96 outpatients/month (59 men and 37 women; 70 ± 11 years). The mean HbA1c level of outpatients improved from 7.7 ± 1.8% to 6.7 ± 0.7% (*P* < 0.001).

**Discussion**

To facilitate medicine teams, physicians need to negotiate in and out of the hospital setting. Accordingly, medical staff's attitude toward diabetes treatment will improve, and thus, they will provide a higher quality of care.

**Conclusions**

Effective diabetes guidance may be provided if an appropriate treatment system is established by a diabetologist.

### AII‐P‐39

#### Hyperglycemia from a rare case of ectopic ACTH‐secreting medullary thyroid carcinoma

**A. F. Lumbera Jr^1^, A. Ceralde^2^, P. D. Maningat^1^ and A. M. Dominguez^3^**

^1^Section of Endocrinology, Diabetes and Metabolism, Department of Medicine, University of the Philippines – Philippine General Hospital^2^Department of Medicine, University of the Philippines – Philippine General Hospital^3^Section of Nephrology, Department of Medicine, University of the Philippines – Philippine General Hospital

**Case**

A young female was admitted for behavioral changes and weight loss. Capillary and random serum glucose were high. She was also hypertensive. Physical exam showed nodular goiter, hyperpigmentation, and lower extremity weakness. Workup revealed hypokalemia, metabolic alkalosis, and normal C‐peptide and HbA1c. Cushing's syndrome was considered due to high cortisol, which was unsuppressed after high dose dexamethasone. ACTH was also high. Imaging was unremarkable. Goiter workup showed normal thyroid function and positive ACTH, calcitonin, and thyroglobulin staining, confirming ACTH‐secreting medullary thyroid cancer.

**Discussion**

Hyperglycemia is a prominent manifestation of Cushing's syndrome. A comprehensive workup is essential.

### AII‐P‐40

#### Newly identified mutation in 5′UTR of HNF1A in a case of MODY3

**Y. Kitamura^1^, N. Iwasaki^2,3,4^, H. Akagawa^3^, M. Ogata^2^, K. Saito^3,4^ and Y. Uchigata^2^**

^1^Internal Medicine, Fujisawa Shonandai Hospital^2^Diabetes Center, Tokyo Women's Medical University^3^Tokyo Women's Medical University Institute for Integrated Medical Science (TIIMS)^4^Institute for Medical Genetics, Tokyo Women's Medical University

HNF1A mutations are the cause of MODY3, in which because of the decreased expression levels of HNF4A and SGLT1/SGLT2, insulin secretion is disrupted and urinary glucose excretion is enhanced.

A 22‐year old woman who had urine sugar pointed out by school medical examination at the age of 14 and had shown impaired glucose tolerance on 75 gOGTT at the age of 19. We found 6 nucleotides (GGGGTT) insertion mutation in the 5′UTR of HNF1A. CentridFold predicted it may form a new stem‐loop structure and IPknot revealed that this structural change may affect the translational efficiency and decrease protein expression of HNF1A. We observed a significant reduction in expression of the mutant allele compared to that in wild type in the reporter assay.

### AII‐P‐41

#### Persistent hypoglycemia in a patient with giant phyllodes tumor successfully treated with dexamethasone and surgery

**J. P. P. Pacaira**

Section of Endocrinology, Diabetes and Metabolism, University of the Philippines‐Philippine General Hospital

A 34‐year‐old pregnant Filipino female presented with a giant phyllodes tumor (PT) of the right breast. She had persistent hypoglycemia episodes despite continuous glucose infusion and increased carbohydrate intake. Investigations pointed to a non‐islet cell tumor hypoglycemia (NICTH). Hypoglycemia improved with Dexamethasone therapy prior to right total mastectomy with split thickness skin grafting. Histopathology of the right breast confirmed a benign PT. Hypoglycemia did not recur after tumor removal. NICTH from PT is a rare cause of hypoglycemia. Treatment involves tumor removal after hypoglycemia is controlled. Glucocorticoids may be used to rapidly induce hyperglycemia via its insulin antagonistic effects prior to definitive therapy.

### AII‐P‐42

#### Sodium glucose cotransporter 2 combined with Cetuximab attenuated multiple liver metastatic colon cancer

**S. Okada and M. Yamada**

Division of Endocrinology and Diabetes Gunma University Hospital

A 69‐year old male patient had been regularly visiting our hospital for the management of sigmoid colon cancer with multiple liver metastases. Because he developed postprandial hyperglycemia, we arranged the dosage of his insulin and added dapagliflozin. Immunohistochemistry study confirmed that sigmoid colon cancer cells express SGLT2. Because cancer cells became resistant against chemotherapies, monotherapy of Cetuximab was started. One month later, not only his CEA level reduced to 112.4 from 1104 (ng/mL) but also multiple metastatic tumor lesions were dramatically shrunken up according to the computed tomography examination. This patient suggests us the possibility of drug repositioning of SGLT2 inhibitors as a cancer treatment.

### AII‐P‐43

#### Pancreatic tuberculosis mimicking pancreatic malignancy in a 33‐year old male with diabetes

**A. J. Sarmiento**

Section of Endocrinology, Diabetes and Metabolism, Department of Medicine, University of the Philippines – Philippine General Hospital

A 33‐year old male was admitted for obstructive jaundice secondary to pancreatic head mass, rule out malignancy. The patient was diagnosed with diabetes mellitus 3 years prior to this admission manifesting as anorexia and generalized weakness. Surgical plan for the patient was exploratory laparotomy, pancreatoduodenectomy. Intraoperative findings revealed multiple nodules on liver, pancreas and omentum. Frozen section was done on respective implants revealing chronic granulomatous inflammation with caseation necrosis and Langhans‐type giant cells, compatible with a tubeculous etiology. Quadruple anti‐ TB medications were started and patient improved.

### AII‐P‐44

#### Relationship between serum lipocalin 2 levels and visceral adiposity in Chinese men

**Y. Bao^1^, Y. Luo^1^, X. Ma^1^, X. Pan^1^, Y. Xu^1^, Q. Xiong^1^, Y. Xiao^2^ and W. Jia^1^**

^1^Department of Endocrinology and Metabolism, Shanghai Jiao Tong University Affiliated Sixth People's Hospital, Shanghai Clinical Center for Diabetes, Shanghai Key Clinical Center for Metabolic Disease, Shanghai Diabetes Institute; Shanghai Key Laboratory o^2^Department of Radiology, Shanghai Jiao Tong University Affiliated Sixth People's Hospital

Serum lipocalin 2 (LCN2) plays an important role in the regulation of the obesity‐associated dysmetabolic state and cardiovascular disease. We aimed to examined the associations of total body fat content and abdominal fat distribution with serum LCN2 levels in 1203 Chinese men aged 22–78 years from the Shanghai Obesity Study. Subjects with a high fat% had higher serum LCN2 levels than those with a normal fat%. A trend of increasing VFA was observed with increasing serum LCN2 levels. VFA was positively associated with serum LCN2 levels, independent of overall obesity and other confounding factors. Serum LCN2 levels were positively associated with not only total body fat but also visceral fat area in Chinese men.

### AII‐P‐45

#### Bile acid profiles in gestational diabetes mellitus

**F. Liu^1^, W. Hou^1^, X. Meng^2^, A. Zhao^1^, W. Zhao^1^ and J. Pan^1^**

^1^Department of Endocrinology & Metabolism, Shanghai Jiao‐Tong University Affiliated Sixth Peoples Hospital^2^Department of Obstetrics and Gynecology, Shanghai Jiao‐Tong University Affiliated Sixth Peoples Hospital

The aim of the current study is to clarify serum bile acid metabolism alteration gestational diabetes mellitus (GDM). a targeted metabolomics study was performed to explore the changes in serum bile acid metabolism of GDM (*n* = 131)and control pregnant women (*n* = 138). The results demonstrated women with GDM had higher lithocholic acid (LCA) levels compared with healthy subjects. LCA was independently associated with GDM. In conclusion, LCA was an independent risk factor of GDM.

### AII‐P‐46

#### Japanese BMI cut‐off points to screen for type 2 diabetes: a 5‐year follow‐up study

**Y. Nakajima^1^, A. Katano‐Toki^1^, M. Akuzawa^2^, K. Sakamaki^2^, T. Satoh^1^, Y. Shimomura^2^, I. Kobayashi^2^, K. Andou^2^ and M. Yamada^1^**

^1^Department of Medicine and Molecular Science, Gunma University Graduate School of Medicine^2^Hidaka Hospital

**Aims**

The ADA has recommended a lower BMI cut‐off point (CO) to screen for T2DM in Asian Americans. The objective of is to confirm age and gender specific BMI CO in Japanese individuals using 5,370 participants.

**Results**

The BMI CO for diabetes within 5 years was 23 kg/m^2^ for men aged <45 with OR of 1.35, and BMI was not a risk factor aged ≥60 men. BMI CO for women were 19 kg/m^2^ with OR of 4.09 for those aged <45 and 20 kg/m^2^ with OR of 1.92 for those aged 45–59 years, and BMI was not a risk factor for women aged ≥60.

**Conclusions**

A BMI CO of 23 kg/m^2^ was appropriate for Japanese men aged <45, whereas a lower BMI may be more suitable for women. BMI was not a risk factor for the development of diabetes in men or women aged ≥60.

### AII‐P‐47

#### Association of osteocalcin with glucose metabolism in the Chinese population

**D. Yan, C. Hu and W. Jia**

Department of Endocrinology, Shanghai Jiao Tong University Affiliated Six People's Hospital

**Aims**

We aimed to evaluate the association of osteocalcin with glucose metabolism.

**Methods**

We recruited 945 males and 4,259 postmenopausal females from Shanghai area. Associations between osteocalcin and glucose metabolism were evaluated.

**Results**

In females, osteocalcin was associated with T2DM (OR [95% CI]: 0.952 [0.931; 0.972]; *P* = 5.2E‐06), insulin sensitivity and insulin secretion. In males, osteocalcin was correlated with HOMA‐β (β ± SE: 0.3044 ± 0.0688; *P* = 1.00E‐05), GUTT‐ISI (β ± SE: 0.2056 ± 0.0570; *P* = 0.0003) and Stumvoll first‐phase insulin secretion index (β ± SE: 0.2322 ± 0.1026; *P* = 0.0240).

**Conclusions**

Osteocalcin was highly associated with T2DM, insulin sensitivity and insulin secretion in both males and postmenopausal females.

### AII‐P‐48

#### Lower hedonic hunger is associated with good glycemic control in obese Chinese patients with type 2 diabetes

**L. T. F. Cheung^1^, G. K. T. Choi^1^, F. C. C. Chung^1^ and A. K. P. Shan^1,2,3^**

^1^Department of Medicine and Therapeutics, The Chinese University of Hong Kong, Prince of Wales Hospital^2^Li Ka Shing Institute of Health Sciences, The Chinese University of Hong Kong^3^Hong Kong Institute of Diabetes and Obesity, The Chinese University of Hong Kong, Hong Kong SAR, China

Hedonic hunger is the drive to eat palatable foods in the absence of physiological need. We explored the association of hedonic hunger and glycemic control in Hong Kong Chinese with T2DM. There were 210 subjects in two groups, non‐obese (BMI 18.5–24) and obese (≥30). Hedonic hunger was assessed by the Power of Food Scale. Good glycemic control (HbA1c <7) was independently associated with male gender, higher BMI, lower waist circumference, DM duration and hedonic drive in the obese group. The odds ratio for an independent association between lower hedonic hunger and good glycemic control was 0.388 (95% CI 0.186–0.810). In conclusion, hedonic hunger was negatively associated with good glycemic control in obese Chinese patients with T2DM.

### AII‐P‐49

#### Triglyceride‐to‐HDL cholesterol ratio is more accurate in detecting metabolic syndrome among middle‐aged Chinese

**Y. Liu^1^, W. Zong^2^, C. Hu^1^, F. Yan^3^, X. Hou^1^, W. Wang^3^ and W. Jia^1^**

^1^Shanghai Diabetes Institute, Shanghai Jiaotong University Affiliated Sixth People's Hospital, Shanghai, China^2^Health Research and Informatics Center of Zhabei district, Shanghai, China^3^Department of Social Medicine and Biostatistics, School of Public Health, Fudan University, Shanghai, China

To assess the accuracy of various lipoprotein fractions and their ratios for detecting metabolic syndrome (MetS) among Chinese, we used the data of 10,640 participants aged 35–60 years old. The two‐stage, stratified sampling survey was conducted from 2009 to 2010. The area under the Receiver Operating Characteristic curve (AUC) and Youden Index were used. Triglyceride/HDL cholesterol (TG/HDL‐C) had the largest AUC of 0.86 (95% CI 0.85–0.87) and 0.82 (95% CI 0.81–0.83) by the criteria of the modified National Cholesterol Education Program's Adult Treatment Panel III and the International Diabetes Federation definition 2005 respectively. TG/HDL‐C was more accurate than other lipid measurements in primary risk screening of MetS among Chinese.

